# Proximity labelling reveals VPS13C as a regulator of *Salmonella-*containing vacuole fission

**DOI:** 10.1371/journal.ppat.1013507

**Published:** 2025-09-15

**Authors:** Anna K. Waldmann, Dustin A. Ammendolia, Andrew M. Sydor, Ren Li, Jonathan St-Germain, Brian Raught, John H. Brumell

**Affiliations:** 1 Cell & Systems Biology, Hospital for Sick Children, Toronto, Canada; 2 Department of Molecular Genetics, University of Toronto, Toronto, Canada; 3 Princess Margaret Cancer Centre, University Health Network, Toronto, Canada; 4 Department of Medical Biophysics, University of Toronto, Toronto, Canada; 5 Institute of Medical Science, University of Toronto, Toronto, Canada; 6 SickKids IBD Centre, Hospital for Sick Children, Toronto, Canada; University of Virginia School of Medicine, UNITED STATES OF AMERICA

## Abstract

*Salmonella enterica* serovar Typhimurium (*S.* Typhimurium) is a facultative intracellular bacterial pathogen that grows within a specialized membrane-bound compartment known as the *Salmonella*-containing vacuole (SCV). The molecular composition and regulatory mechanisms governing SCV dynamics remain incompletely understood. In this study, we employed proximity-dependent biotin identification (BioID) to analyze the SCV proteome during infection. For this, we targeted the UltraID biotin ligase to the SCV by fusing it to a type 3 secreted effector. We demonstrate that the bacteria express and translocate the effector-UltraID fusion protein directly into host cells for labeling of the cytosolic face of the SCV surface. Proteomic analysis of biotinylated proteins revealed previously undescribed proteins associated with the SCV, including regulators of vesicular trafficking, cellular metabolism and lipid transport. Among these, VPS13C, a lipid transporter and membrane contact site protein, was identified as a critical regulator of SCV morphology and fission. Functional studies revealed that VPS13C also promotes ER-SCV contact formation, controls SCV positioning in host cells, and facilitates cell-to-cell spread by the bacteria. Together, our findings highlight the utility of BioID as a tool to study host-pathogen interactions in the context of infection and characterize VPS13C as a novel modulator of the intracellular life cycle of *S.* Typhimurium.

## Author summary

*Salmonella enterica* serovar Typhimurium (*Salmonella*) is a bacterium present in our food supply that can cause food poisoning. These bacteria can also cause severe disease in immunocompromised individuals, young children and the elderly. An important feature of *Salmonella* is that it can invade the cells of its host during infection. Once inside host cells, *Salmonella* resides within a membrane-bound compartment known as the *Salmonella*-containing vacuole (SCV), where it can replicate and evade immune defenses. The SCV is a dynamic structure that interacts with various host cell components, but the full range of these interactions, and how they contribute to generating a vacuolar niche for bacterial replication remains poorly understood. In this study, we used a new enzyme-based labeling strategy to identify host cellular proteins on the SCV during infection. This approach identified VPS13C, a lipid transporter not previously linked to *Salmonella* infection. We found that VPS13C localizes to the SCV and is important for maintaining its structure and promoting bacterial spread between cells. These findings provide new insights into how *Salmonella* exploits host cell machinery and demonstrate a powerful method for studying the life cycle of pathogen-containing vacuoles in the context of infection.

## Introduction

Non-typhoidal *Salmonella* serovars are pathogens with both epidemic and pandemic potential and have been designated as priority pathogens for research by the World Health Organization [1]. This includes *Salmonella enterica* serovar Typhimurium (*S.* Typhimurium), a Gram-negative, facultative intracellular bacterial pathogen with a global distribution [2]. These bacteria have also served as an important model pathogen for the study of Salmonellosis [2,3].

A hallmark of *S.* Typhimurium’s intracellular lifecycle is the simultaneous bacterial cell division and fission of the *Salmonella*-containing vacuole (SCV) [4,5]. This process predominantly results in SCVs containing one or two dividing bacteria, with only about 4% of SCVs harbouring three or more bacteria [6]. Despite its importance in pathogenesis, the mechanisms underlying SCV fission remain incompletely understood. Previous studies have implicated host factors such as Dynein, Dynactin [6], Syntaxin-3 [7], RAB7, PLEKHM1 and the HOPS complex [8] in this process. Emerging evidence suggests a potential role for the endoplasmic reticulum (ER) in SCV fission [9]. This is supported by observations of ER contact with SCV membranes, facilitated by the lipid transfer protein OSBP [10]. Additionally, bacterial virulence proteins (called effectors) that are translocated into host cells by type 3 secretion systems (T3SS) are known to modify the SCV [11]. Notably, the T3SS secreted effector proteins SteA (*Salmonella* translocated effector A) and SopD2 (*Salmonella* outer protein D2) have been shown to be critical for initiating SCV fission [5], though the precise mechanisms by which they act to regulate SCV fission are unknown.

Previous attempts to characterize SCVs have employed centrifugation-based organelle fractionation and SCV membrane enrichment, including precipitation of SCVs from various mammalian cell types and *Dictyostelium discoideum* [12–15]. Although these methods have proven insightful, they are labour-intensive and rely on harsh lysis conditions which can hinder the detection of weak or transient interactions. Membrane contact sites (MCS), now appreciated to play an important role in the generation of pathogen-containing vacuoles [16–19], are likely disrupted during such SCV isolation approaches. Furthermore, SCV isolation approaches provide only a static snapshot of the interactions at the time of vacuole isolation. Proximity-dependent biotin identification (BioID) has been used to examine the host-pathogen interface in other cellular contexts [20] and offers a promising alternative to examine the SCV composition during infection.

Classic BioID utilizes a modified version of the *Escherichia coli* biotin ligase BirA (BirA*), fused to a protein of interest (bait) [21]. BirA* generates a “cloud” of the chemical intermediate biotinoyl-AMP, which reacts with lysine residues within a ~ 10 nm radius [22]. These biotinylated proteins can then be captured using streptavidin and identified by mass spectrometry [23]. BioID has previously been applied to study *S.* Typhimurium effectors in the absence of infection by overexpressing effector-BirA* fusions in mammalian cell lines under normal growth conditions [24]. While this method revealed a number of infection-relevant host targets, it does not capture interactions that arise due to host cell rewiring upon bacterial infection.

Ideally, performing BioID with effector-BirA* fusions generated by bacteria and translocated into host cells to associate with the SCV would enable proteomic analysis of this compartment during infection. However, translocation of T3SS effectors is often impaired by their fusion to large or folding-sensitive proteins [25,26]. A new biotin ligase variant, UltraID [27], possesses increased biotinylation activity and is approximately half the size of BirA*, making it an attractive candidate for T3SS-compatible secretion.

Here, we perform BioID during *S.* Typhimurium infection using an effector-UltraID bait to examine the SCV proteome. Our approach demonstrates the utility of BioID using the UltraID tool to study host-pathogen interactions in the context of infection. In doing so, we characterize VPS13C as a proximal interactor of the SCV with relevance to SCV fission and *Salmonella* cell-to-cell spread.

## Materials and methods

### Cell culture

HeLa cells were obtained from the American Type Culture Collection (ATCC). U2-OS cells were a gift from Dr. Peter Kim (SickKids, Toronto). All parent cell lines were tested for *Mycoplasma* upon receipt from manufacturer, and results were negative. Cells were maintained in high-glucose DMEM (Wisent, 319–005 CS, lot 319005528) supplemented with 10% FBS (Wisent, #090–450, lot 112755) at 37°C with 5% CO_2_. For microscopy-based studies, cells were seeded in 24-well tissue culture plates containing 12 mm glass coverslips at a concentration of 4.5 x 10^4^ cells/well 24 h before use. Transfections were performed using PolyJet (SignaGen, #SL100688, lot 61778) according to the manufacturer’s instructions, or by electroporation with the P3 Primary Cell 4D-Nucleofector X Kit L (Lonza, #V4XP-3024).

### CRISPR knockout (KO) lines

To disrupt specific gene expression in HeLa cells and U2-OS cells, human-specific single-guide RNAs (sgRNA) were designed using the online tool https://chopchop.cbu.uib.no. Custom sgRNA oligonucleotides were synthesized by Sigma Aldrich.

For VPS13C, the sgRNA sequences used were: #1, 5’- CACCGTTCATACCAATGGTCGACGA-3’ and 5’- AAACTCGTCGACCATTGGTATGAAC-3’; #2, 5’- CACCGAACTTACCATCGTCGACCAT-3’ and 5’- AAACATGGTCGACGATGGTAAGTTC -3’; #3, 5’- CACCGTTTAATAGGGCTACGAATA-3’ and 5’- AAACTATTCGTAGCCCTATTAAAC-3’. sgRNA sequences were introduced into the BbsI site of pX459 the CRISPR/Cas9 vector pSpCas9 (BB)-2A-Puro (pX459) (Addgene plasmid #62988) [28] and the constructs were verified by DNA sequencing (TCAG, Toronto). Cells were then transfected with the ligated vector and 24h later the transfected cells were selected by puromycin (2 µg/ml) for another 48 h. Cells transfected with the empty pX459 vector were used as control cells. Single cells were then transferred into a 96-well plate and allowed to grow until confluent; knockout efficiency was validated by western blot.

### Bacterial strains and infections

Infections were performed with wild-type *S*. Typhimurium SL1344 or 14028S and isogenic mutants lacking the effectors of interest or the SPI-2 T3SS component SsaR [29].

A previously established approach was used for infection of epithelial cells [30] using late-log *S.* Typhimurium cultures as inocula. Briefly, sub-cultured *Salmonella* strains were pelleted at 10,000 x *g* for 2 min, resuspended and diluted 1:100 in PBS, pH 7.4, and added to cells for 10 min at 37°C. Selection for intracellular bacteria was performed at 30 min p.i. using 100 µg/ml gentamicin, a concentration that was decreased to 10 µg/ml at 2 hours post infection. When applicable, cells were fixed with 2.5% paraformaldehyde in PBS at 37°C for 15 min.

### Gentamycin protection assay

HeLa cells were infected with *Salmonella* strains as above. At 30 min, 2 h, 4 h, 6 h, 8 h, 10 h and 24 h post-infection, infected host cells were washed three times with PBS, lysed in PBS, pH 7.4, with 1.0% Triton X-100 and plated as a dilution series on LB agar supplemented with streptomycin (50 µg/ml). After incubation at 37°C, colony forming units (CFU) were counted. Each time point was performed in triplicate, and each individual experiment was performed at least three times.

### Plasmids

VPS13C^mClover3 was a gift from Pietro De Camilli (Addgene plasmid #118760) [31]. VPS13C mutants were created using site directed mutagenesis. VPS13C^mClover3 (A444P) was generated using the following primers: 5’-ccaaggcaacaagcacaagttgaggtgattc-3’ and 5’-taaaattatgttaaaaacatctagagtcttc-3’. VPS13C^mClover3 (W395C) was created using 5’-tgc agtaacataaaaaagcacaggcagttactc-3’ and 5’-tgaccacatctgtgtataccttcttatatg-3’.

SopD2-HA/pACYC184 was previously described [32]. SopD2-UltraID-HA/pACYC184 was generated via restriction cloning. SopD2-HA/pACYC184 was digested with XhoI and BamHI. UltraID was amplified from pet-15b-ultraID using 5’- GATCATCTCGAGgacttcaagaacctgatctggctgaag-3’ and 5’-GATCATGGATCCTTACGCATAATCCGGCACATCATACGGATACGCATAATCCGGCACATCATACGGATActtctccttgaacttcttcaggttctc-3’, introducing the corresponding restriction sites.

pet-15b-ultraID was a gift from Julien Béthune (Addgene plasmid #172879) [27] SopD2-HA/pCOND was created via restriction cloning. XbaI and SbfI cut sites were introduced with 5’-GATCATTCTAGATTTAAGAAGGAGATATACATATGCCAGTTACGTTAAGTTTTGGTAATCGTC-3’ and 5’-GATCATCCTGCAGGGACCACACCCGTCCTGTGGATCCTTACGCATAATCCG-3’. pCON1-ProD.gfp was a gift from Olivia Steele-Mortimer (Addgene plasmid #112518) [33].

SopD2-HA/pACYC184 and SopD2-UltraID-HA/pACYC184 were used as templates for PCR. GFP was excised from pCON1-ProD.gfp using XbaI and SbfI. A synthetic linker (amino acid sequence: AAAAAAAAAAAAYKHIATTRLFAAAAAAAAAAAAAAAAAAAAAAAAAAAAAAAAAAAA, was introduced between SopD2 and UltraID via inverse PCR. UltraID-2HA was subcloned into a CMV-driven Clontech GFP backbone by Gibson assembly. The UltraID-2HA insert was PCR-amplified from the SopD2-UltraID-HA template using primers (5′-tcagatctcgagccaccatggacttcaagaacctgatctggctgaaggag-3′ and 5′-ctagagtcgcggccgctttaTTACGCATAATCCGGCACATCATACGGATACGCAT-3′) that provided vector overlaps for assembly. mCherry-P4M-SidM was a gift from Tamas Balla (Addgene plasmid # 51471 [34]. All constructs were transformed into NEB 5-alpha competent E. coli (NEB, #C29921, lot 10228973). DNA sequences were verified by DNA sequencing (The Centre for Applied Genomics, Toronto) or whole plasmid sequencing (Eurofins).

### *Salmonella* infection and biotinylation for BioID

Δ*sopD2* SL1344 was complemented with SopD2-UltraID-HA by electroporation. Subsequently, HeLa cells were infected with Δ*sopD2* SL1344 expressing SopD2-UltraID-HA. Biotin labelling efficiency was assessed between 10 – 24 hours post-infection by immunofluorescence. HeLa cells were seeded in four 150 mm dishes at a concentration of 15 x 10^6^ cells 24 h before use. Infections were performed as outlined above, with exception that biotin (BioBasic, #BB0078, lot O5B13DA1) was added to infection media at a final concentration of 50 μM for 23 hours.

### BioID sample preparation

Cell pellets were resuspended in 10 ml of Lysis Buffer (50 mM Tris-HCl pH 7.5, 150 mM NaCl, 1 mM EDTA, 1 mM EGTA, 1% Triton X-100, 0.1% SDS, 1:500 protease inhibitor cocktail (Sigma-Aldrich), 1:1000 benzonase nuclease (Novagen) and incubated at 4°C for 1 hour. Following sonication lysates were cleared by centrifugation (30 minutes,16,000 x *g,* 4°C). Supernatants were transferred to a fresh 15 ml conical tube, and 30 μl streptavidin sepharose beads (GE) were added. Samples were incubated for 3 hours at 4°C with end-over-end rotation. Beads were rinsed 6 times with 1 ml of 50 mM ammonium bicarbonate (NH_4_HCO_3_, pH = 8.3). Tryptic digestion (1 μg MS-grade TPCK trypsin (Promega, Madison, WI)) was performed overnight at 37°C. The following morning, 0.5 μg of trypsin was added, and beads were incubated 2 additional hours at 37°C. Supernatants were collected, beads were rinsed with NH_4_HCO_3_ (50 mM) and eluates were pooled. The sample were desalted using C18 tips, lyophilized and resuspended in 0.1% HCOOH prior to LC-MS analysis.

### LC-MS

Digested, de-salted and lyophilized peptides were reconstituted in 0.1% HCOOH and 500 ng (as measured by absorbance at 205 nm) were loaded onto Evotips (Evosep, Odense Denmark). Liquid chromatography was performed using the Evosep One (Evosep, Odense Denmark) pump with an SPD30 method using an Evosep Performance C18 HPLC column (15 cm x 150 µm ID, 1.5 µm; Evosep, Odense Denmark). The TIMS-TOF HT (Bruker, Bremen) mass spectrometer was operated in PASEF-DDA positive ion mode (MS scan range 100–1700 m/z). Ion mobility range was 1/K_0_ = 1.6 to 0.6 Vs cm^-2^ using equal ion accumulation and ramp time in the dual TIMS analyzer of 100 ms each. Collision energy was lowered stepwise as a function of increasing ion mobility, starting from 20 eV for 1/K_0_ = 0.6 Vs cm-2 and 59 eV for 1/K_0_ = 1.6 Vs cm^-2^. The ion mobility was calibrated linearly using three ions from the Agilent ESI LC/MS tuning mix (m/z, 1/K_0_: 622.0289, 0.9848 Vs cm-2; 922.0097, 1.1895 Vs cm-2; and 1221.9906, 1.3820 Vs cm^-2^). Data files were analyzed on the Fragpipe (v22.0) using MSFragger (v4.1). A merged database containing the Human Uniprot and *S.* Typhimurium (22,544 total entries) databases and including reversed-sequence decoys was searched with trypsin as protease (2 missed cleavage allowed), and acetylation (protein N-term), oxidation (M), and deamidation (NQ) as variable modifications. Search results were validated using the Percolator algorithm (v3.6.5) platform. All raw data are available through the MassIVE repository under accession MSV000098876 [35].

### Immunofluorescence staining

Cells were fixed with 2.5% paraformaldehyde in PBS for 15 min at 37°C. Immunostaining was performed as previously described [29] using the following primary antibodies: rabbit monoclonal anti-HA (Cell Signaling Technology, #3724, lot 10) at a dilution of 1:200, mouse monoclonal anti-LAMP-1 (DSHB, #H4A3-s, lot 11/1/18) at a dilution of 1:200, goat polyclonal anti-SCAMP3 (Santa Cruz, # sc-13624) at a dilution of 1:200, rabbit polyclonal anti-*Salmonella* (BD Transduction, #229481, lot 4017189) at a dilution of 1:1000, rabbit polyclonal anti-GFP (Invitrogen, #A11122, lot 2339829) at a dilution of 1:200.

The following Alexa Fluor (AF)-conjugated secondary antibodies were used in this study: AF488-conjugated goat anti-mouse IgG (Invitrogen, #A-11029, lot 2179204) and anti-rabbit IgG (Invitrogen, #A-11034, lot 2541675), AF568-conjugated goat anti-mouse IgG (Invitrogen, #A-11031, lot 2026148) and anti-rabbit IgG (Invitrogen, #A-11011, lot 2379475), AF647-conjugated goat anti-mouse IgG (Invitrogen, #A-32728, lot XE344349) and anti-rabbit IgG (Invitrogen, #A-32733, lot TL272452). Biotin was detected using AF-568-conjugated streptavidin (Invitrogen, #S11226, lot 2045314). All secondary antibodies were used at a dilution of 1:500 and host cell nuclei were stained using DAPI (2 µg/ml).

### *Salmonella* containing vacuole positioning

Following immunostaining for LAMP1 and *Salmonella*, as well as DAPI staining, images of infected cells were acquired. SCV-positioning relative to the nearest edge of the host cell nucleus was determined using Volocity 6.3 software (Quorum Technologies Inc). SCVs were identified as LAMP1^+^ vacuoles containing *Salmonella*. Every SCV was measured in at least 10 infected cells, such that greater than 50 bacteria were assessed per biological replicate of the experiment.

### Cell-cell spread assay

Cell-to-cell spread by *S*. Typhimurium was examined as previously described [35]. Fluorescently labeled, uninfected HeLa cells were seeded over a layer of unlabeled, infected cells (primary infected cells) in 24-well tissue culture plates. This permitted examination of whether intracellular bacteria could migrate into the newly introduced uninfected cells (secondary infected cells). HeLa cells (5 × 10^4^ cells/well) were infected with WT SL1344 and Δ*ssaR* (SPI-2 secretion deficient). To fluorescently label secondary HeLa cells, a flask of HeLa cells (80–90% confluence) was washed three times in PBS and incubated in serum-free DMEM containing 25 μM CellTracker Blue (Molecular Probes, #C211, lot 1756357) for 45 min. Cells were thoroughly washed three times with PBS and allowed to recover in DMEM supplemented with 10% fetal bovine serum for 30 min. At 2 hours p.i., CellTracker Blue-labeled HeLa cells were seeded over the previously infected cells at two times the original density (10 × 10^4^ cells/well). Cells were fixed at 10 hours p.i. and 24 hours p.i. and immunostained for *Salmonella* and LAMP1 and visualized by fluorescence microscopy. LAMP1^+^ SCVs found within secondary cells were regarded as evidence for cell-to-cell infection. All experiments were conducted in the presence of gentamicin (10 μg/ml) to inhibit invasion of HeLa cells by any extracellular bacteria. 100 secondary cells surrounding primary infected cells were counted and assessed for presence of LAMP1^+^ SCVs per biological replicate.

### Confocal microscopy

Cells were imaged using a Quorum spinning disk microscope (Quorum) with a 63x oil immersion objective (Leica DMIRE2 inverted fluorescence microscope equipped with a Hamamatsu Back-Thinned EM-CCD camera or Hamamatsu CMOS FL-400 camera, spinning disk confocal scan head) and Volocity 6.3 acquisition software (Improvision). Confocal *z*-stacks of 0.3 μm were acquired, and images were analyzed with Volocity 6.3 software.

### Transmission electron microscopy

Cells were fixed in 2% paraformaldehyde and 2.5% glutaraldehyde in 0.1 M sodium cacodylate buffer for 2 hours at room temperature (RT), followed by incubation at 4°C. After fixation, samples were washed three times for 10 minutes each in 0.1 M sodium cacodylate buffer at RT. Post-fixation was performed with 2% osmium tetroxide and 1% potassium ferrocyanide in 0.1 M sodium cacodylate buffer for 2 hours at RT. Samples were then washed three times with distilled water for 10 minutes each and stained with freshly filtered thiocarbohydrazide (TCH) for 30 minutes at RT. After another series of three 10-minute washes in distilled water, samples were incubated in 2% osmium tetroxide in distilled water for 1 hour at RT, followed by three additional distilled water washes. For en bloc staining, samples were incubated in 2% aqueous uranyl acetate overnight at 4°C, then washed three times with distilled water. Lead aspartate staining was performed for 1 hour at 60°C using freshly prepared solution, followed by three final washes in distilled water at RT. Samples were dehydrated through a graded ethanol series (50%, 70%, 90%, and three changes of 100% ethanol, each for 15 minutes at RT), followed by three 10-minute washes in propylene oxide. Infiltration was performed with a graded series of Epon 812 resin in propylene oxide: 1:1 for 2 hours, 1:2 for 2 hours, followed by two changes of 100% Epon 812 for 2 hours each at RT. Samples were then incubated in fresh 100% Epon 812 overnight at RT or for 8 hours. Finally, samples were embedded in fresh resin and polymerized for 48 hours at 65°C.

### SCV contact analysis

Transmission electron microscopy (TEM) analysis was performed on samples from three biological replicates. Quantitative analysis was carried out on randomly selected 2D EM sections, focusing on membrane contact sites between the SCV and the ER. Membrane contact was defined as <45 nm [36]. For each image, masks for SCVs, ER, and mitochondria were manually generated in FIJI. These binary masks were used for contact analysis with a custom Python script adapted from the DeepContact pipeline [37], modified for 2D analysis using FIJI-derived masks. The script calculates distance maps and identifies contact regions based on spatial proximity between labeled compartments. A minimum of 30 SCVs per replicate was analyzed for both control and VPS13C knockout HeLa cells. Quantified metrics included: (i) SCV area; (ii) total SCV contact with ER and mitochondria; (iii) number of ER contact points per SCV (normalized to SCV area); and (iv) percentage of SCV membrane in contact with the ER. Source code of the script located: https://github.com/anna-waldmann/membrane-contact-analysis

### Western blotting

Cell lysates were resolved by 10% SDS-PAGE, transferred to PVDF membrane (Bio-Rad), and probed with antigen-specific primary antibodies. The following primary antibodies were used for western blot detection: mouse monoclonal anti-HA (Covance, #MMS-101R, lot EI2BF00286), rabbit polyclonal anti-VPS13C (Proteintech, #28676–1-AP, lot 00092796) rabbit polyclonal anti-GFP (Invitrogen, #A11122, lot 2339829), mouse monoclonal anti-beta-actin (Sigma, # A5441, lot 0000137632). Primary antibodies were used at a dilution of 1:1000. Blocking and staining was performed with 5% skim milk in TBS-T (20 mM Tris,150 mM NaCl, 0.1% Tween 20). For all antibody-based analyses, horseradish peroxidase (HRP)-conjugated secondary antibodies were used: goat anti-rabbit IgG (Jackson ImmunoResearch, #11-035-144, lots 152081 and 163676), goat anti-mouse IgG (Jackson ImmunoResearch, #111-035-146, lot 157140). Biotinylation was assessed using StrepTactin-HRP (Bio-Rad, #161–0380, lot L004034A). Secondary antibodies were used at a dilution of 1:2000. Detection was performed using Clarity Western ECL Substrate (BioRad, #1705060, lot 102032080) or SuperSignal West Femto Maximum Sensitivity Substrate (Thermo, #34095, lot ZO388314) as required, and results were analyzed using Image Lab v6.1 (BioRad).

### Statistics

For all studies, a minimum of three independent experiments were performed, and the mean ± SD is shown in the figures. *P*-values were calculated with GraphPad Prism v10.2.1 using a two-way ANOVA with Tukey’s test, unless otherwise indicated. A *p*-value < 0.05 was determined to be statistically significant with annotations as follows: *p* < 0.05 (*), *p* between 0.001 and 0.01 (**), **p* *< 0.001 (***), and **p* *< 0.0001 (****). Where applicable, n.s. represents a comparison that is not statistically significant (**p* *> 0.05).

## Results

### BioID analysis of the SCV membrane proteome

To investigate the membrane proteome of SCVs during infection, we employed proximity-dependent biotin identification (BioID). For this, we targeted UltraID, the smallest biotin ligase currently available [27], to the SCV by fusing it to a T3SS effector. Thus, bacteria expressed and translocated the effector-UltraID fusion protein directly into host cells for labeling of the cytosolic face of the SCV surface.

**Fig 1:**
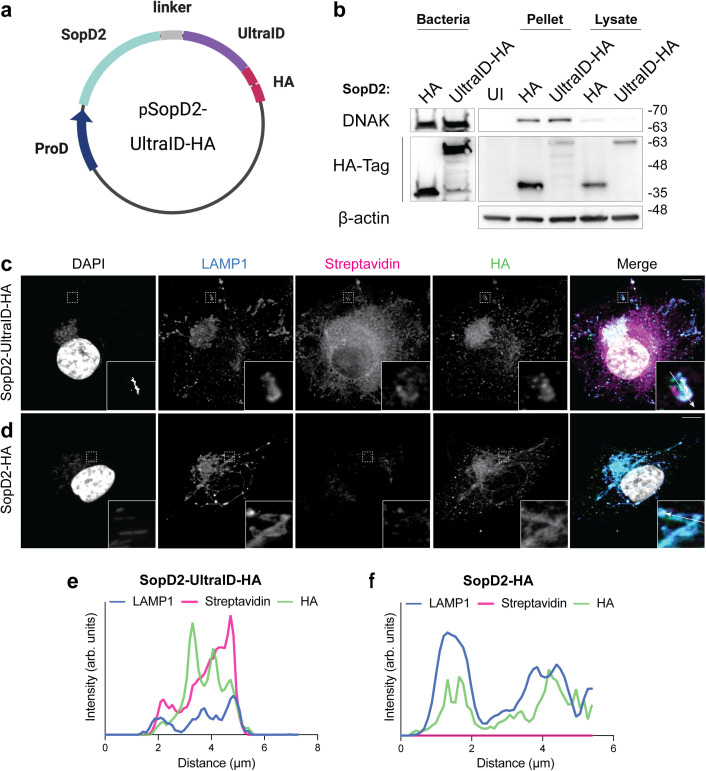
UltraID fusion to SopD2 enables biotin labeling of the SCV. Representative images are shown and the associated scale bars for fluorescence images indicate 10 μm. a, Effector-UltraID construct expressed from the pCON-proD vector system. SopD2 was C-terminally fused to a GS-linker followed by UltraID and a tandem HA-tag. b, Western blot of bacterial and infected host cell lysate. For infection sub-cultured strains expressing SopD2-UltraID-HA or SopD2-HA were pelleted and boiled in SDS. Infected cell lysate was harvested 23 h p.i.. c, HeLa cells infected with a Δ*sopD2* mutant of *S.* Typhimurium SL1344 expressing SopD2-UltraID-HA, d, HeLa cells infected with *S.* Typhimurium expressing SopD2-HA (control). Cells in (c) and (d) were fixed 23 h p.i. and stained for LAMP1, HA-tag and Biotin (Streptavidin 568 probe); insets show regions with *S.* Typhimurium inside LAMP1^+^ SCVs. Contrast enhancement of the DAPI channel was performed in the insets displayed to highlight *S.* Typhimurium. e, Line plot profile of the white arrow in the inset of the merged image in (c). In this and following panels, arb. units (arbitrary units) indicate the signal densities along the chosen white arrow, f, Line plot profile of the white arrow in the inset of the merged image in (d).

We utilized SopD2, a SPI-2 T3SS effector known to decorate SCVs [32] for targeting of UltraID during infection. SopD2 was fused to the N-terminus of UltraID and expressed from a low-copy selectable plasmid (Fig 1a). Two hemagglutinin epitope tags (-HA) were fused to the C-terminus of UltraID to enable analysis of the expression and localization of the fusion protein using HA antibodies. To enhance expression throughout the infection timecourse, SopD2-UltraID-HA was constitutively expressed downstream of a synthetic promoter in the pCON-proD vector [33]. To further improve the translocation efficiency from bacteria to the host cell cytoplasm, a flexible synthetic linker [38] was introduced between SopD2 and UltraID (Fig 1a).

The plasmid encoding SopD2-UltraID-HA was transformed into a Δ*sopD2* mutant of *S*. Typhimurium SL1344 [32]. In bacterial cultures grown to late logarithmic phase we observed expression of the SopD2-UltraID-HA fusion protein that was comparable to our control plasmid expressing SopD2 with two C-terminal HA tags (SopD2-HA) [32] (Fig 1b). These bacteria were then used to infect HeLa cells for 23 hours (h), a timepoint sufficient for SopD2 translocation into host cells and association with SCVs [32]. Under these conditions, we observed efficient translocation of SopD2-UltraID-HA into HeLa cells as judged by western blotting of infected host cell lysates (Fig 1b) and immunofluorescence analysis with HA antibodies (Fig 1c and 1d). SopD2-UltraID-HA and SopD2-HA localized to SCVs (Fig 1c and 1d; see insets) and other LAMP1^+^ compartments, consistent with prior studies [32]. SopD2 is known to contribute to the formation of *Salmonella*-induced filaments (SIFs), tubular extensions of the SCV [32,39]. Expression of SopD2-UltraID-HA was sufficient to complement formation of *Salmonella*-induced filaments (SIFs) in the Δ*sopD2* mutant of *S*. Typhimurium, indicating that the function of SopD2 was not compromised by its fusion to UltraID (S1a and S1b Fig).

Next, we examined the ability of the SopD2-UltraID fusion construct to perform biotinylation reactions during infection. For this, biotin was added to the extracellular medium throughout a 23 h infection to allow for continuous labeling of the SCV cytoplasmic surface and its interactions with other organelles during infection. Using Streptavidin-AF568 as a probe for biotinylated proteins, we observed a robust signal in cells infected by bacteria expressing SopD2-UltraID-HA (Fig 1c and 1e). Biotinylated proteins were observed to colocalize with SCVs and other cellular compartments. In contrast, robust biotinylation (i.e., above ‘background’ signal observed in uninfected cells) was not observed in cells infected with the control SopD2-HA plasmid (Fig 1d and 1f). Thus, our findings indicate that SopD2-UltraID-HA is effectively expressed by bacteria and translocated into host cells where it has enzymatic activity.

Having established that the fusion of SopD2 to UltraID properly translocates, localizes and functions, we proceeded to conduct a full BioID experiment. HeLa cells were infected with bacteria expressing SopD2-UltraID-HA for 23 h with biotin present in the extracellular medium. We selected 23 h post-infection for proximity labeling, as this late stage of infection represents a time when SCVs accumulate in host cells, thereby maximizing the likelihood of detecting specific SCV-associated proteins. Although SopD2-UltraID-HA expression was detectable throughout the infection time course (S1c Fig), 23 h was chosen because it represents a biologically optimal stage for labeling.

**Fig 2:**
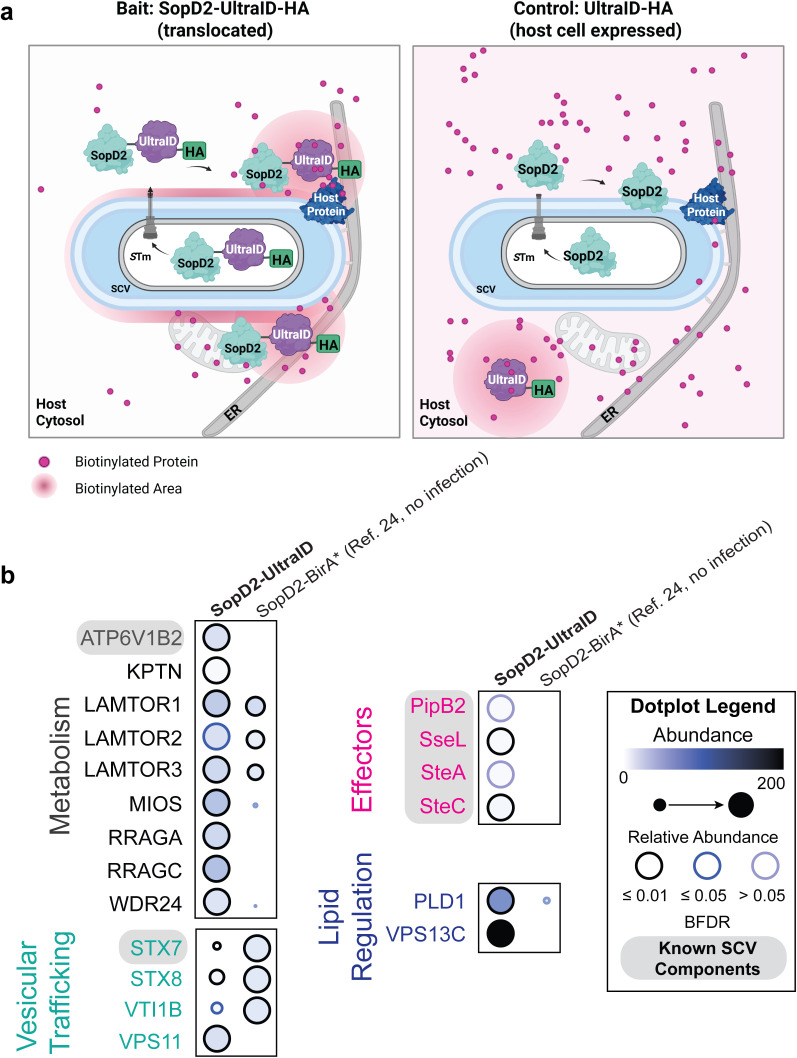
Proteomic analysis reveals VPS13C as a proximal member of the SCV. a, Schematic of SCV biotin labeling with a Type 3 Secreted effector. *S*. Typhimurium expresses and translocates the effector-UltraID fusion protein directly into host cells for labeling of the cytosolic face of the SCV surface. To control for background biotinylation, an enzyme-only control (UltraID-HA) was ectopically expressed in the host cell cytosol. Created in BioRender. Waldmann. A. (2025) https://BioRender.com/0mwigcy. b, The ProHits-viz web tool was used to generate the dot plot view highlighting known SCV components and novel SCV proximity interactors from the BioID dataset, displaying prey abundance and prey confidence (BFDR). BioID data generated with SopD2-UltraID-HA was compared to previous data for SopD2-BirA* from D’Costa *et al,* 2019 [24]. Dot size and color intensity represent prey protein abundance (total spectral counts), while dot outline color reflects BFDR significance (black ≤ 0.01, blue ≤ 0.05, light blue > 0.05). Relative abundance is normalized to the highest abundance for each prey across all baits.

As a negative control we expressed UltraID-HA in host cells, which were subsequently infected with *S*. Typhimurium SL1344. As illustrated in Fig 2a, SopD2-UltraID-HA is translocated into host cells via the SPI-2 T3SS, whereas the control, UltraID-HA, is ectopically expressed in the host cytosol. Biotin was present throughout the 23-hour infection period to match the labeling time of SopD2-UltraID. Immunofluorescence confirmed that UltraID-HA localized to the cytosol and nucleus, but not to SCVs (S2a and S2b Fig) despite higher levels of UltraID-HA expression compared to SopD2-UltraID-HA (S2c and S2d Fig).

Overall, we identified 87 high-confidence proximity interactors for SopD2-UltraID-HA (S1 Table). Gene ontology analysis was performed on these interactors, revealing significant enrichment of proteins involved in vesicular trafficking, lipid regulation, and cellular metabolism (Fig 2b). This enrichment aligns with previous studies that have characterized the composition of isolated SCVs and *Salmonella*-modified membranes [12–15]. Several of our high-confidence hits were previously shown to localize to SCVs and have distinct roles in *S*. Typhimurium infection, including Syntaxin 7 (STX7) [40] and several components of the vATPase, including ATP6V1B2 [41]. We also identified several SPI-2 T3SS effectors known to decorate SCVs, namely SteA [9], SteC [42], PipB [43] and SseL [10]. The identification of these known SCV-associated proteins indicated that SopD2-UltraID-HA serves as an effective tool for probing the SCV membrane proteome.

### VPS13C is a component of the SCV membrane proteome

In our BioID dataset, we identified VPS13C, a lipid transporter located at ER-late endosome/lysosome contact sites [31]. Unlike lipid exchangers of the ORP family (e.g., OSBP) which transfer one lipid at a time, VPS13 proteins function as bulk lipid transporters, facilitating the flow of lipids between membranes at MCS [31,44]. To our knowledge, this study represents the first report linking VPS13C to host-pathogen interactions.

### VPS13C regulates SCV morphology

**Fig 3:**
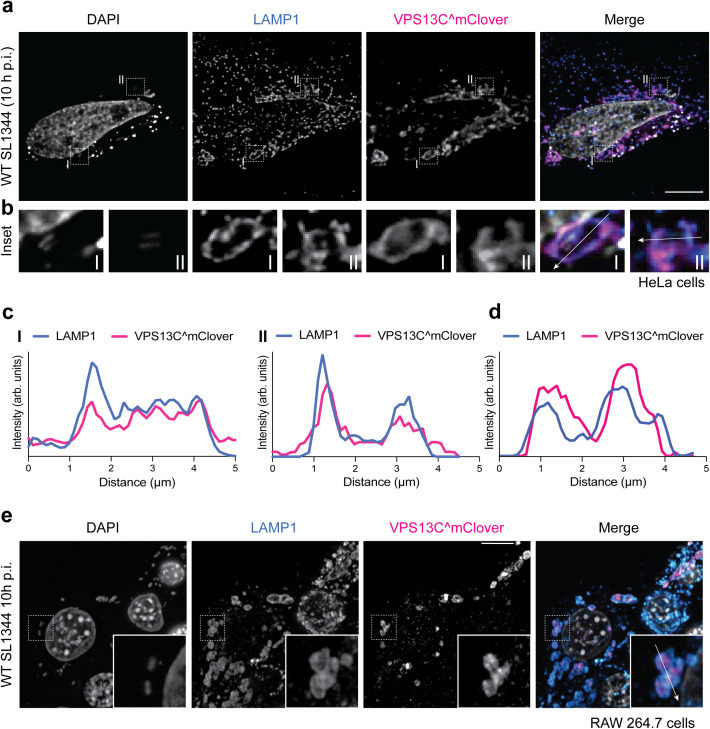
VPS13C localizes to SCVs in different cell types. Representative images are shown, and the associated scale bars for fluorescence images indicate 10 μm. a, HeLa cells transfected with VPS13C^mClover and infected with *S.* Typhimurium. Cells were fixed at 10 h p.i. and immunostained for LAMP1. VPS13C signal was boosted with a GFP antibody. DAPI was used for DNA staining (nuclei and *S.* Typhimurium). b, Insets (I and II) show regions with *S.* Typhimurium inside LAMP1^+^ SCVs, colocalizing with VPS13C^mClover. c, Line plot profile of the white arrow in the insets (I and II) of the merged image in (a,b). d, Line plot profile of the white arrow in the inset of the merged image in (e). e, RAW 264.7 macrophages transfected with VPS13C^mClover and infected with *S.* Typhimurium. Cells were fixed at 10 h p.i. and immunostained for LAMP1. VPS13C signal was boosted with a GFP antibody. DAPI was used for DNA staining (nuclei and *S.* Typhimurium)*.*

To explore the potential involvement of VPS13C in *Salmonella* pathogenesis, we examined the localization of VPS13C internally tagged with mClover3 at residue 1914 (VPS13C^mClover) [31] in HeLa cells. In uninfected cells, VPS13C^mClover localized to LAMP1^+^ structures (S3a and S3b Fig), consistent with previous reports [31]. Upon *S*. Typhimurium SL1344 infection of HeLa cells, VPS13C^mClover was enriched at SCVs 10 h p.i. (Fig 3a–3c). We also observed VPS13C^mClover association with SCVs in RAW264.7 macrophages (Fig 3d and 3e) as early as 2 h p.i., with localization persisting through 24 h p.i. (S4a and S4b Fig), indicating that recruitment to SCVs is not cell type specific. VPS13C recruitment to SCVs was not SPI-2-effector-induced, as VPS13C^mClover still localized to SCVs in HeLa cells infected with a SPI-2 T3SS mutant at 10 h p.i (S3c Fig). We did not detect an interaction between SopD2 and VPS13C using two different co-immunoprecipitation strategies (S3d and S3e Fig). First, we co-expressed VPS13C^mClover and SopD2-RFP and performed a pulldown with GFP-trap beads (S3d Fig). In a second approach, we used endogenous VPS13C immunoprecipitated with anti-VPS13C antibodies and protein G beads, while overexpressing only SopD2-RFP (S3e Fig). In both cases, SopD2-RFP was not detected in the bound fraction. These results are consistent with previous studies that also did not detect VPS13C among SopD2 interactors by co-IP [45]. Together, these findings suggest that while co-IP is not well suited to capture this interaction, our BioID approach can identify novel proteins that localize to or associate with SCVs.

**Fig 4:**
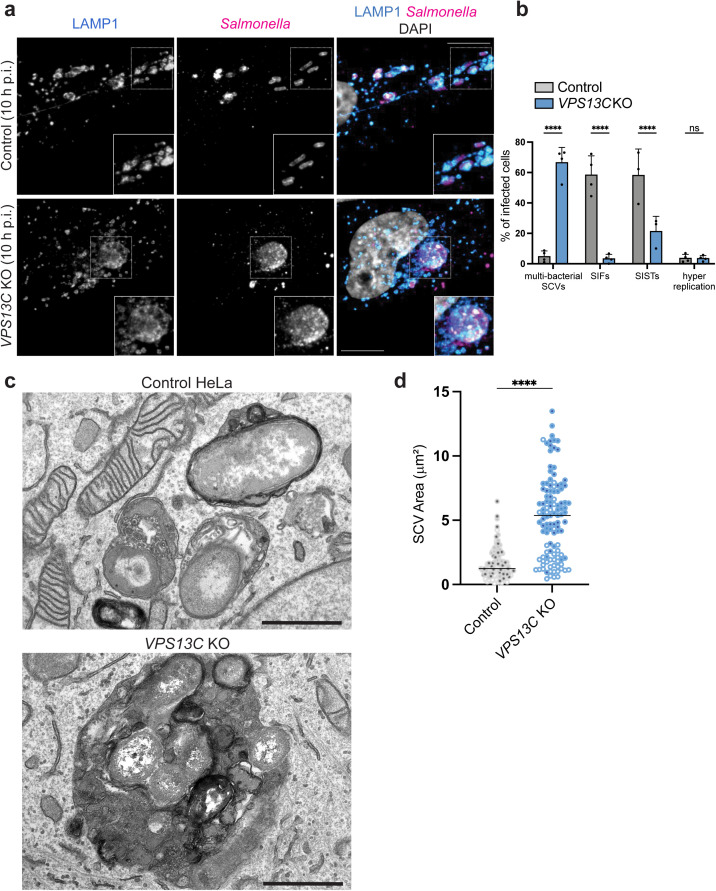
VPS13C is required for the regulation of SCV morphology and fission. Representative images are shown and the associated scale bars for fluorescence images indicate 10 μm. a, *VPS13C* KO HeLa and control cells infected with *S.* Typhimurium and stained for LAMP1 and *Salmonella* 10 h p.i.. b, Quantifications of (a), 100 infected cells assessed for presence of SIFs, SISTs, multi-bacterial SCVs and hyper replication; *p* value was calculated using two-way analysis of variance (ANOVA) (n = 4). c, Representative transmission electron micrograph (TEM) of an infected *VPS13C* KO HeLa cell and control cell. Scale bars indicate 1 μm. d, Quantification of SCV size. SCV area (μm²) was measured from binary masks with a custom Python script; ≥ 30 SCVs per condition per experiment. Each circle is a single SCV, with fill shades denoting independent experiments (n = 3).

We next generated *VPS13C* knockout (KO) HeLa cells using CRISPR and confirmed loss of VPS13C protein expression by western blotting (S5a Fig). Control and knockout cells were then infected with *S*. Typhimurium and analyzed at various timepoints ranging from 10 - 23 h p.i.. A hallmark of the *S.* Typhimurium intracellular lifecycle is the simultaneous bacterial cell division and fission of the SCV [4,5], creating multiple SCVs per cell that contain one or two bacteria on average [6]. In *VPS13C* KO cells, we observed the formation of larger SCVs (herein referred to as “multi-bacterial SCVs”) containing more than two bacteria per vacuole at 10 h p.i. (Fig 4a and 4b). Multi-bacterial SCVs were consistently observed across three independent *VPS13C* KO HeLa cell clones (S5a and S5b Fig). Additionally, *Salmonella*-induced tubule formation (LAMP1^+^ SIFs and SCAMP3^+^ SISTs, see ref [46]), was significantly reduced in *VPS13C* KO cells compared to infected control cells (Fig 4b). Notably, these observations were not limited to HeLa cells as multi-bacterial SCVs were also observed during *S.* Typhimurium infection of U2-OS cells lacking VPS13C expression (S6a and S6b Fig). Multi-bacterial SCV formation was also observed with a different background of *S.* Typhimurium (WT 14028S), ruling out strain-specificity of the multi-bacterial SCV phenotype in *VPS13C* KO HeLa cells (S6c–S6e Fig). Translocation of effectors (PipB2, SifA, SopD2) into host cells and their association with SCVs was observed in *VPS13C* KO cells (S7a and S7b Fig), indicating that expression and activity of the SPI-2 T3SS was not affected by loss of VPS13C.

### VPS13C contributes to ER-SCV contact formation

We used transmission electron microscopy (TEM) to further examine *VPS13C* KO cells during infection, observing that multi-bacterial SCVs had a single limiting membrane enclosing multiple bacteria (Fig 4c). SCVs in control cells averaged <1 μm², whereas multi-bacterial SCVs averaged >5 μm² (Fig 4d). These findings suggest that VPS13C is required for scission of the SCV, an event normally coupled to bacterial cell division [4,6,9].

**Fig 5:**
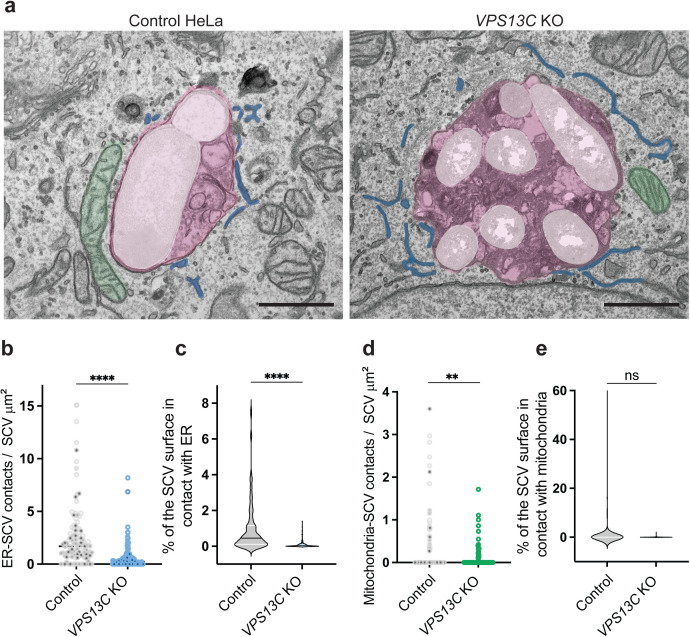
VPS13C contributes to ER-SCV contact formation. a, Representative images of random 2D TEM sections of *VPS13C* KO and control HeLa cells. Regions relevant to SCV contact analysis were false-colored: SCVs (magenta), *S*. Typhimurium (white), mitochondria (green), and ER (blue). Scale bars indicate 1 μm. b, Number of ER–SCV (a) or mitochondria-SCV (b) contacts per SCV normalized to SCV surface. Contact was defined as ER membrane (a) or mitochondria membrane (b) within <45 nm of the SCV membrane in 2D sections. SCV and ER masks were drawn in FIJI and analyzed with a custom Python script (adapted from DeepContact [37]) to count discrete contact sites for each SCV. Each circle is one SCV, with fill shades denoting independent experiments (n = 3). At least 30 SCVs were analyzed per condition per experiment. Groups were compared with a two-tailed unpaired t-test. c, Percent of SCV surface area in contact with ER (c) or mitochondria (d). For each SCV, the area of SCV surface within <45 nm of ER (nm²) was divided by total SCV area (nm²) and expressed as percent coverage. Violin plots show the distribution across SCVs pooled from three independent experiments, with internal lines indicating median and quartiles. At least 30 SCVs were analyzed per condition per experiment. Groups were compared with a two-tailed unpaired t-test. d, Number of mitochondria-SCV contacts per SCV normalized to SCV surface. Contact was defined as mitochondria membrane within <45 nm of the SCV membrane in 2D sections. Each circle is one SCV, with fill shades denoting independent experiments (n = 3). At least 30 SCVs were analyzed per condition per experiment. Groups were compared with a two-tailed unpaired t-test. e, Percent of SCV surface area in contact mitochondria. For each SCV, the area of SCV surface within <45 nm of mitochondria (nm²) was divided by total SCV area (nm²) and expressed as percent coverage. Violin plots show the distribution across SCVs pooled from three independent experiments, with internal lines indicating median and quartiles. At least 30 SCVs were analyzed per condition per experiment. Groups were compared with a two-tailed unpaired t-test.

Because VPS13C serves as a membrane tether between the ER and other organelles [31,47], we used TEM to analyze SCV-organelle contacts, defining contacts as regions of membrane apposition separated by <45 nm [36,48]. Regions used for contact analysis are highlighted in false-colored TEM images (Fig 5a). For each SCV, we quantified both the number of discrete ER-SCV and mitochondria-SCV contacts (Fig 5b and 5d) as well as the fraction of the SCV perimeter in contact with ER or mitochondria (Fig 5c and 5e). Both the number of ER-SCV contacts and the percentage of SCV area in contact with the ER were reduced in *VPS13C* KO cells compared to controls. While we observed a slight reduction in mitochondria-SCV contact number in *VPS13C* KO cells (Fig 5d), the percentage of SCV area engaged with mitochondria was not significantly different (Fig 5e). Together, these data support a role for VPS13C in sustaining ER-SCV interactions [46].

Phosphatidylinositol 4-phosphate (PI4P) is known to be dephosphorylated by an ER resident phosphatase (SAC1) at sites of membrane contact with other organelles [49]. Therefore, we hypothesized that the altered ER-SCV contacts observed in *VPS13C* KO cells could impact levels of PI4P on the SCV. To test this, we examined the SCV using a biosensor based on the P4C domain of the *Legionella pneumophila* effector protein SidC, which specifically binds PI4P [50]. Using this PI4P biosensor, we observed PI4P decorated SCV membranes at 10 h p.i. in control cells and multi-bacterial SCVs in *VPS13C* KO cells. Quantification of PI4P intensity at SCVs, normalized to LAMP1, showed a significant increase of PI4P signal in the absence of VPS13C (S8a and S8b Fig). Some signal may originate from intraluminal vesicles, consistent with TEM evidence of vesicular accumulations within multi-bacterial SCVs, yet the overall increase together with reduced ER-SCV contacts supports a model in which limited ER engagement in VPS13C KO cells restricts PI4P clearance at the SCV.

### PD-associated *VPS13C* variants fail to rescue the multi-bacterial SCV phenotype in *VPS13C* KO cells

**Fig 6:**
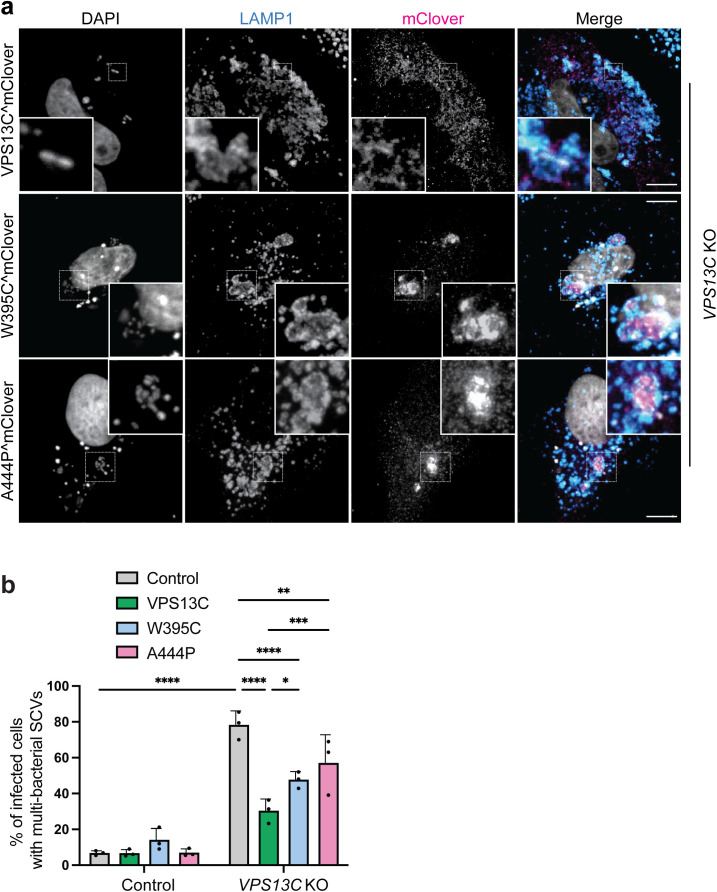
PD-associated variants of VPS13C fail to rescue the multi-bacterial SCV phenotype in *VPS13C* KO cells. Representative images are shown and the associated scale bars for fluorescence images indicate 10 μm. a, *VPS13C* KO HeLa cells were transfected with VPS13C^mclover, A444P-VPS13C, W395C-VPS13C or control plasmid and infected with *S.* Typhimurium. Cells were fixed 10 h p.i. and immunostained for LAMP1. VPS13C signal was boosted by staining with a GFP antibody. DAPI was used for DNA staining (nuclei and *S.* Typhimurium). b, Quantification of SCV size. SCV area (μm²) was measured from binary masks with a custom Python script; ≥ 30 SCVs per condition per experiment. Each circle is a single SCV, with fill shades denoting independent experiments (n = 3).

To assess whether the multi-bacterial SCV phenotype observed in *VPS13C* KO cells could be rescued upon complementation, we expressed VPS13C^mClover in these cells and performed infections with *S.* Typhimurium (Figs 6a and S9a). Following infection, we quantified the proportion of transfected and infected cells that exhibited the multi-bacterial SCV phenotype. Expression of VPS13C^mClover partially rescued the phenotype, with a significant reduction in the number of multi-bacterial SCVs compared to knockout cells transfected with an empty vector construct (Figs 6b and S9a).

Homozygous and compound heterozygous mutations in the VPS13C gene have been implicated in the development of early-onset Parkinson’s disease (PD) [51]. Two compound heterozygous missense mutations in *VPS13C*, W395C and A444P, are associated with autosomal recessive early-onset Parkinson’s disease (PD) [51,52]. Modeling with AlphaFold revealed that these mutations are present in the N-terminal domain of VPS13C (S10a Fig). AlphaFold modeling of VPS13C carrying the W395C or A444P mutations showed only minor local rearrangements in the N-terminal helical cluster (S10b and S10c Fig), though the impact of these substitutions remains uncertain given the limitations of AlphaFold in predicting the consequences of point mutations.

PD has been associated with defects in lysosomal and mitochondrial function [53], and VPS13C has been implicated in maintaining homeostasis of these organelles [54–56]. Given the role of VPS13C in SCV regulation, we reasoned that PD-associated mutations might impair its function during *S.* Typhimurium infection. Therefore, we evaluated the complementation efficiency of VPS13C mutants associated with PD by expressing these mutants in the *VPS13C* KO HeLa cells (Figs 6a and S9a). Expression of VPS13C^mclover-A444P and VPS13C^mclover-W395C in *VPS13C* KO HeLa cells was less effective at rescuing the multi-bacterial SCV phenotype compared to WT VPS13C^mClover, as a higher proportion of transfected cells continued to exhibit multi-bacterial SCVs (Figs 6a, 6b, and S9a). Therefore, the tested PD-associated mutations impair the ability of VPS13C to restore normal SCV morphology and suggest relevance of these mutations in VPS13C-mediated processes.

### VPS13C is required for *Salmonella* cell-to-cell spread

**Fig 7:**
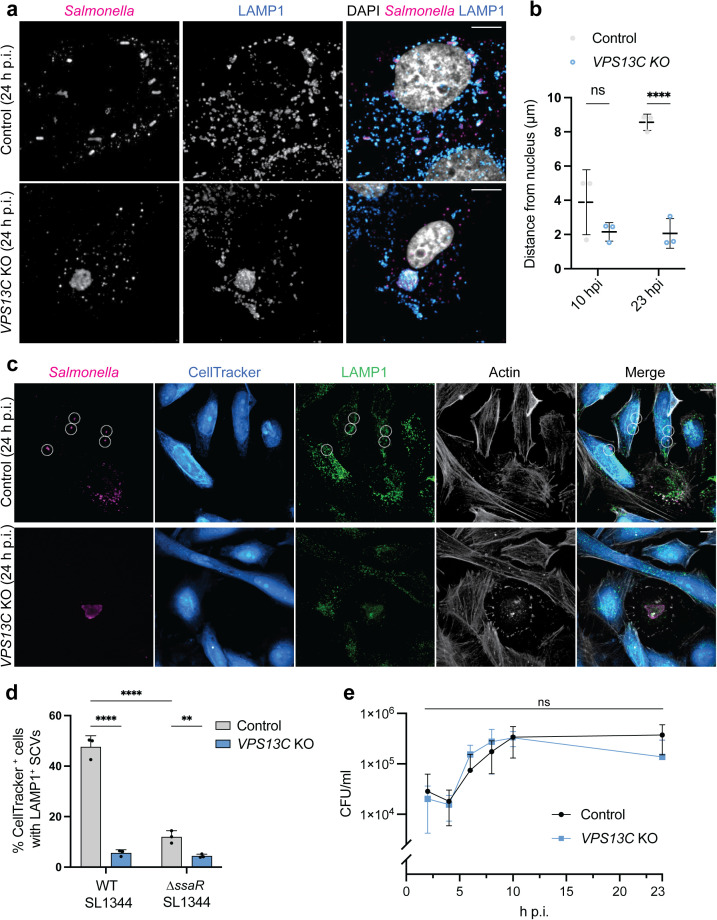
VPS13C is required for *Salmonella* cell-to-cell spread. Representative images are shown and the associated scale bars for fluorescence images indicate 10 μm. a, *VPS13C* KO HeLa and control cells infected with *S.* Typhimurium. Cells were fixed 24 h p.i. and stained for LAMP1 and *Salmonella*. b, Distribution of SCVs in *VPS13C* KO HeLa cells and control cells relative to the host cell nucleus at 10 h and 23 h p.i.. The distances of LAMP1 + SCVs to the nearest edge of the host cell nucleus were measured. The averages of three independent experiments are shown in the graph. Data are presented as means ± S.D. The *P* value was calculated using two-way ANOVA (n = 3). c, HeLa cells were infected with *S.* Typhimurium and fixed at 23 h p.i.. Cells were immunostained for *Salmonella* (white), LAMP1 (green), and actin (magenta). Secondary cells were stained with CellTracker Blue. Dotted circles in (c) indicate LAMP1^+^ SCVs in secondary cells. d, Percent of CellTracker Blue-labeled HeLa cells from the above experiment that contained LAMP1^+^ SCVs with WT SL1344, and SPI-2 deficient (*ΔssaR*). The averages ± standard deviations for three independent experiments are shown. One hundred CellTracker Blue-labeled HeLa cells were counted for each experiment. The *P* value was calculated using two-way ANOVA (n = 3). e, Total intracellular bacterial CFUs were assessed by gentamicin protection assay in *VPS13C* KO HeLa cells and control cells. Data are shown as means ± S.D. The *P* value was calculated using a two-tailed unpaired t-test. No significant differences were observed.

SCVs undergo a dynamic distribution within host cells during the bacterial intracellular infection cycle. At intermediate timepoints of infection (8–14 h p.i.), SCVs typically assume a perinuclear position and are often associated with the Golgi network [35,57,58]. Subsequently, SCVs undergo a centrifugal displacement toward the host cell periphery (14–24 h p.i.) in a manner that requires the SPI-2 T3SS and host microtubule motors [35,59]. Centrifugal movement of SCVs has been shown to promote cell-to-cell spread by *S.* Typhimurium [35,59]. Of relevance here, we observed that multi-bacterial SCVs generated in *VPS13C* KO HeLa cells remained perinuclear at late stages (23 h) of infection (Fig 7a).

To characterize the role of VPS13C in SCV positioning, we measured the distance of LAMP1^+^ SCVs from the nearest edge of the host cell nucleus. SCVs were observed in the perinuclear region at 10 h p.i. in both control and *VPS13C* KO HeLa cells (Fig 7b). At 23 h p.i., SCVs were positioned closer to the plasma membrane (~8 μm from the nearest nuclear edge) in control cells (Fig 7a and 7b), consistent with prior observations [35,59]. In contrast, SCVs remained within ~2 μm of the nearest nuclear edge at 23 h p.i. in *VPS13C* KO cells (Fig 7a and 7b). Thus, VPS13C deficiency prevents centrifugal movement of SCVs at late post infection times.

These findings suggested that loss of VPS13C might impair cell-to-cell spread of *S.* Typhimurium. To test this hypothesis, we performed cell-to-cell spread assays following the protocol described by Szeto *et al*. [35]. In brief, cells infected with *S.* Typhimurium were overlaid with an uninfected, labelled population (referred to as secondary cells) and incubated for an additional 24 h. Cell-to-cell spread was then assessed through detecting bacteria in LAMP1^+^ SCVs in secondary cells. As a negative control, primary cells were infected with a Δ*ssaR* mutant, which is deficient in cell-to-cell spread [35,59].

In experiments with control cells, we observed that ~47% of CellTracker-labeled secondary cells contained LAMP1^+^ SCVs (Fig 7c and 7d), indicative of cell-to-cell spread by *S.* Typhimurium. In contrast, only ~5% of secondary cells harbored SCVs in *VPS13C* KO cell cultures (Fig 7c and 7d). Minimal bacterial spread was observed in both control and *VPS13C* KO cells following infection with Δ*ssaR* mutant bacteria, as expected [35,59]. To determine whether the reduction in *S.* Typhimurium spread in *VPS13C* KO cells was due to differences in intracellular growth, we conducted gentamicin protection assays. We observed similar intracellular growth rates for *S.* Typhimurium in *VPS13C* KO and control cells (Fig 7e), indicating that the lack of cell-to-cell spread in *VPS13C* KO cells was not due to impaired intracellular growth. Thus, our findings suggest a critical role for VPS13C in regulating SCV dynamics that allow for cell-to-cell spread.

## Discussion

Our findings indicate that BioID analysis of the SCV membrane proteome can be achieved during *S*. Typhimurium infection. Our bait protein, SopD2-UltraID-HA, was successfully expressed, translocated by the *S*. Typhimurium T3SS, and localized to SCVs in infected cells. This enabled continuous biotin labeling of SCVs during infection and led to the identification of several previously unreported proteins as candidate interactors of the SCV membrane.

We identified VPS13C as a proximal interactor of the SCV, where it influences vacuole morphology and ER contacts, and contributes to *S*. Typhimurium cell-to-cell spread. Our findings suggest that membrane contact sites (MCS) formed with the ER enable lipid flow for SCV fission and/or microtubule motor recruitment. Previous work demonstrated that the lipid transport protein OSBP is recruited for SCV maintenance [10] and PDZD8 was recently identified as an SCV-localized tethering protein [45]. Together with our identification of VPS13C, these observations highlight that multiple ER-SCV tethers converge at this interface, although direct exploitation of these factors by *S*. Typhimurium has not been demonstrated. A useful parallel can be drawn to the PITT pathway [60], where ATG2 functions as a lipid transporter that enables subsequent recruitment of additional tethering factors. Analogously, VPS13C may function as a primary ER–SCV bridge, facilitating the action of other transporters and tethering proteins. This model is supported by our TEM data, which revealed reduced ER contacts with SCVs in the absence of VPS13C. Other intracellular pathogens are also known to exploit multiple MCS during colonization of host cells [16–19]. However, the nature of lipid flow to pathogen-containing vacuoles and the molecular mechanisms regulating this flow remain unclear. MCS are likely to also have other roles in controlling SCV morphology. For example, ER-SCV contact sites were recently proposed to facilitate SCV fission through a rapid contact-dependent process [9].

VPS13C mutations have been associated with several biological traits and pathologies, most notable Parkinson’s disease [51,52]. It is noteworthy that PD-associated mutations have been linked to host protection from *S*. Typhimurium and other pathogens [61,62]. Similarly, we found that expression of PD-associated VPS13C mutants (W395C and A444P) [51,52] was less effective at rescuing the multi-bacterial SCV phenotype in *VPS13C* KO cells compared to the wildtype gene. These findings suggest that these PD-associated mutations impair the function of VPS13C in regulating SCV morphology, further supporting the functional relevance of these mutations in VPS13C-mediated processes. Whether PD-associated mutations in VPS13C impact host-pathogen dynamics in human populations is not clear. However, it is noteworthy that other PD-associated mutations in other genes have been linked to host protection from *S*. Typhimurium and other pathogens [61,62].

Our approach reveals BioID as a powerful tool to study host-pathogen interactions during infection. There are, however, limitations to this approach and the application of BioID to other intracellular pathogen-containing vacuoles will require careful bait design: i) Tolerance of tagging. Prior knowledge that SopD2 targets to SCVs in host cells and can tolerate fusion to epitope tags [32] helped us select this T3SS effector as a fusion partner for UltraID and we anticipate these features will be important for bait design with other pathogens. ii) Expression levels. We used a synthetic promoter to enhance expression of the SopD2-UltraID-HA fusion protein, an approach that may also be necessary in other pathogens. iii) Linker length. It will also be important to test different linkers for effector-UltraID fusions. For *S*. Typhimurium infection, we determined that the insertion of a long linker (58 amino acids) between SopD2 and UltraID was critical for efficient secretion through the T3SS. Although such a linker worked in our model system, it does have potential issues including alterations to protein domain folding/interactions and increased labeling radius [22,23]. iv) The need for an enzyme-only control. Ideally, such a control - here UltraID-HA- should be secreted into host cells via the SPI-2 T3SS. In our hands this proved challenging, as fusion of UltraID-HA to a type 3 secretion signal was unsuccessful. As an alternative, we ectopically expressed UltraID-HA in host cells, which were then infected with *S.* Typhimurium. While we confirmed that the enzyme itself did not localize to the SCV membrane (a point that should be validated in other models), the large difference in expression levels may have led to the exclusion of bona fide interactors.

Altogether, we present a powerful new technique for examining the proximity interactome of pathogen-containing vacuoles during infection of host cells. We used this method to identify VPS13C as a new host factor in the maintenance of SCV morphology and bacterial cell-to-cell spread. The involvement of the ER in SCV maturation presents a compelling avenue for future research. Our BioID technique and resultant dataset provides a wealth of novel proteins that will serve as the foundation for further investigation into SCV dynamics.

## Supporting information

S1 Fig UltraID-tagging does not compromise SopD2 function. Representative images are shown and the associated scale bars for fluorescence images indicate 10 μm. a, HeLa cells were infected with WT SL1344; a Δ*sopD2* mutant of *S.* Typhimurium SL1344; Δ*sopD2* SL1344 expressing SopD2-UltraID-HA, or Δ*sopD2* SL1344 expressing SopD2-HA. Cells were fixed 10 h p.i. and stained for LAMP1 and HA-tag. DAPI was used for DNA staining (nuclei and *S.* Typhimurium). b, Quantifications of (a), 100 infected cells assessed for presence of SIFs; *P* value was calculated using one-way analysis of variance (ANOVA) (n = 3). c, Time course of SopD2 expression in infected HeLa cells. Western blot analysis comparing SopD2-HA expressed from its native promoter (pACYC184 backbone) with SopD2-UltraID-HA expressed from a synthetic constitutive promoter (pCON-D) at 3–23 h post-infection.
(TIF)

S2 Fig UltraID does not independently localize to SCV membranes. Representative images are shown and the associated scale bars for fluorescence images indicate 10 μm. a, HeLa cells were transfected with UltraID-2HA and infected with *S.* Typhimurium. Cells were fixed 23 h p.i. and immunostained for LAMP1 and HA-tag. DAPI was used for DNA staining (nuclei and *S.* Typhimurium). The line plot profile corresponds to the white arrow in the inset of the merged image. b, HeLa cells were infected with Δ*sopD2* SL1344 expressing SopD2-HA or Δ*sopD2* SL1344 expressing SopD2-UltraID-HA. Cells were fixed 23 h p.i. and immunostained for LAMP1 and HA-tag. DAPI was used for DNA staining (nuclei and *S.* Typhimurium). The line plot profiles correspond to the white arrows in the insets of the merged images. c, HeLa cells were transfected and infected or infected as described in (a) and (b). Cells were fixed 23 h p.i. and stained for HA-tag and Biotin (Streptavidin 568 probe). d, Western blot of infected host cell lysate. HeLa cells were transfected with UltraID-2HA and infected with *S.* Typhimurium or infected with Δ*sopD2* SL1344 expressing SopD2-HA or Δ*sopD2* SL1344 expressing SopD2-UltraID-HA. Cells were harvested 23 h p.i.
(TIF)

S3 Fig VPS13C recruitment is not SPI-2 effector-dependent. Representative images are shown and the associated scale bars for fluorescence images indicate 10 μm. a, HeLa cells transfected with VPS13C^mClover and immunostained for LAMP1. VPS13C signal was boosted with a GFP antibody. DAPI was used for DNA staining (nuclei and *S.* Typhimurium). b, Line plot profile of the white arrow in the inset of the merged images in (a). c, HeLa cells transfected with VPS13C^mClover and infected with Δ*ssaR* SL1344. Cells were fixed 10 h p.i. and immunostained for LAMP1. VPS13C signal was boosted with a GFP antibody. DAPI was used for DNA staining (nuclei and *S.* Typhimurium). d, Immunoprecipitation of VPS13C^mClover from HeLa cells transfected with SopD2-RFP using GFP-trap beads, followed by western blotting for SopD2-RFP. e, Immunoprecipitation of endogenous VPS13C from HeLa cells transfected with SopD2-RFP using anti-VPS13C antibody–conjugated protein G beads, followed by western blotting for SopD2-RFP.
(TIF)

S4 Fig VPS1C is recruited to SCVs in RAW macrophages. Representative images are shown and the associated scale bars for fluorescence images indicate 10 μm. a, RAW 264.7 macrophages were transfected with VPS13C^mClover by electroporation and infected with *S.* Typhimurium. Cells were fixed at 2 h, 6 h,10 h or 24 h p.i. and immunostained for LAMP1. VPS13C signal was boosted with a GFP antibody. DAPI was used for DNA staining (nuclei and *S.* Typhimurium). b, Line plot profile of the white arrow in the inset of the merged images in (a).
(TIF)

S5 Fig Multi-bacterial SCVs are consistently observed across independent *VPS13C* KO HeLa cell clones. Representative images are shown, and the associated scale bars for fluorescence images indicate 10 μm. a: Three different gRNAs and a combination of gRNAs [1–3] were tested for knockout of *VPS13C* in HeLa cells. gRNA 3 and the combination of gRNAs [1–3] were sufficient for knockout of *VPS13C* in HeLa cells. Western blotting was performed to confirm gene knockout. b, Single cell selection was performed on HeLa cells transfected with a combination of gRNAs [1–3]. Clones were expanded and Western blotting was performed to confirm gene knockout. Knockout of *VPS13C* was confirmed in clones #1,#3 and #4. c, *VPS13C* KO clones #1,#3 and #4 and control cells were infected with WT SL1344 and immunostained for LAMP1 and *Salmonella*.
(TIF)

S6 Fig VPS13C-associated multi-bacterial SCV phenotype is observed in other cell types and bacterial strains. Representative images are shown, and the associated scale bars for fluorescence images indicate 10 μm. a: *VPS13C* KO U2-OS and control cells infected with *S.* Typhimurium and immunostained for LAMP1 and *Salmonella*. b: A combination of gRNAs [1–3] was used for sufficient knockout of *VPS13C* in U2-OS cells. *VPS13C* KO U2-OS and control cells were expanded after single-cell selection. Western blotting was performed to confirm gene knockout. Membranes were probed for VPS13C and actin. c,d,e: *VPS13C* KO HeLa cells and control cells were infected with WT SL1344 and WT 14028S. Quantifications of (c,d,e): 100 infected cells were assessed for the presence of multi-bacterial SCVs (c), SIFs (d), and hyper-replication (e). The averages ± standard deviations for three independent experiments are shown. *P* values were calculated using two-way ANOVA (n = 3).
(TIF)

S7 Fig Translocation of SPI-2 T3SS effectors (PipB2, SifA, SopD2) in *VPS13C* KO cells. Representative images are shown and the associated scale bars for fluorescence images indicate 10 μm. a, *VPS13C* KO HeLa cells and control cells were infected with Δ*pipB2* SL1344 expressing PipB2-HA, Δ*sifA* SL1344 expressing SifA-HA, or Δ*sopD2* SL1344 expressing SopD2-HA. Cells were fixed 10 h p.i. and stained for LAMP1, SCAMP3 and HA-tag. DAPI was used for DNA staining (nuclei and *S.* Typhimurium). b, Quantification of (a): a minimum of 50 cells were assessed for the presence of HA signal (PipB2-HA, SifA-HA or SopD2-HA) on SCVs. The averages ± standard deviations for three independent experiments are shown. *P* values were calculated using two-way ANOVA (n = 3).
(TIF)

S8 Fig Limited ER engagement in *VPS13C* KO cells restricts PI4P clearance at the SCV. a, *VPS13C* KO HeLa cells and control cells were transfected with mCherry-P4M-SidM and infected with *S.* Typhimurium. Cells were fixed 10 h p.i. and immunostained for LAMP1 and *Salmonella.* b, Quantification of (a). mCherry-P4M-SidM intensity at the SCV was normalized to LAMP1 for at least 30 SCVs per condition per experiment (n = 3). Conditions were compared with a two-tailed ratio paired t-test.
(TIF)

S9 Fig PD-associated mutations impair the ability of VPS13C to restore normal SCV morphology. Representative images are shown and the associated scale bars for fluorescence images indicate 10 μm. a, Control HeLa cells were transfected with VPS13C^mclover, A444P-VPS13C, W395C-VPS13C or control plasmid and infected with *S.* Typhimurium. Cells were fixed 10 h p.i. and immunostained for LAMP1. VPS13C signal was boosted by staining with a GFP antibody. DAPI was used for DNA staining (nuclei and *S.* Typhimurium).
(TIF)

S10 Fig Predicted structure of VPS13C PD variants. a, The structure of VPS13C predicted by AlphaFold2 [47]. The major domains are indicated as follows: Chorein motif, purple; WD40 modules, magenta; DH-Like domain, blue; Pleckstrin homology domain, orange. The inset is a magnification of the boxed region, which contains the amino acids W395 and A444 (depicted in yellow and blue sticks, respectively). b, AlphaFold3 predicted structures of WT (tan) and W395C (teal) VPS13C (aa 1–1860). Inset is a magnification of the boxed region and depicts stick representations of W395 and C395. c, AlphaFold3 predicted structures of WT (tan) and A444P (teal) VPS13C (aa 1–1860). Inset is a magnification of the boxed region and depicts stick representations of A444 and P444. All AlphaFold3 structural predictions were generated using the same seed and only amino acids 1–1,860, similar to the strategy used by Cai et al. [47]. The Matchmaker tool (Needleman-Wunsch algorithm) in ChimeraX (version 1.10) was used for the structural alignments using the top ranked AlphaFold3 model for each point mutant.
(TIF)

S1 Table BioID analysis of the SCV membrane proteome. Summary of BioID results for SopD2-UltraID-HA compared to UltraID-HA control. The table lists spectral counts (Spec) and summed spectra (SpecSum) for each protein identified, with one sheet showing all detected proteins (unfiltered) and a second sheet showing proteins filtered at BFDR < 0.01.
(XLSX)
