# Correction: Proximity labelling reveals VPS13C as a regulator of *Salmonella-*containing vacuole fission

**DOI:** 10.1371/journal.ppat.1013816

**Published:** 2025-12-30

**Authors:** Anna K. Waldmann, Dustin A. Ammendolia, Andrew M. Sydor, Ren Li, Jonathan St-Germain, Brian Raught, John H. Brumell

In the Plasmids subsection of the Materials and Methods, there is an error in the third sentence of the fourth paragraph. The correct sentence is: A synthetic linker (amino acid sequence: GSSGSSGSSGSSGSSGSSGSSGSSGSSGSSGSSGSSEKSSCRNMLIGSSGSSGSSGSS, was introduced between SopD2 and UltraID via inverse PCR.
