# Supplementary figures and images for: Proximity labelling reveals VPS13C as a regulator of Salmonella-containing vacuole fission
Source: PLoS Pathog. 2025 Sep 15;21(9):e1013507. doi: 10.1371/journal.ppat.1013507 (PMC12492975; doi:10.1371/journal.ppat.1013507)

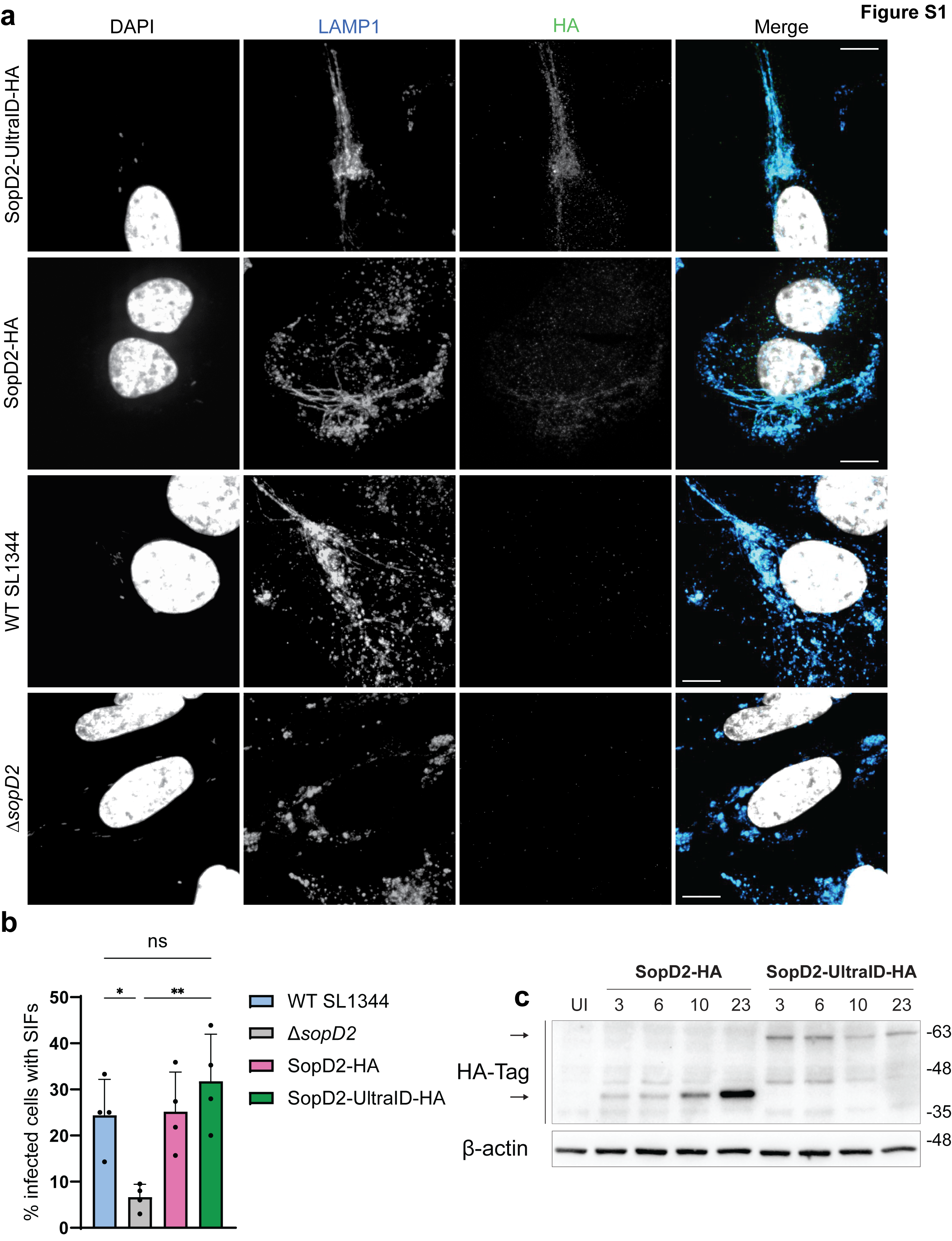

Supplement: S1 Fig — Representative images are shown and the associated scale bars for fluorescence images indicate 10 μm. a, HeLa cells were infected with WT SL1344; a ΔsopD2 mutant of S. Typhimurium SL1344; ΔsopD2 SL1344 expressing SopD2-UltraID-HA, or ΔsopD2 SL1344 expressing SopD2-HA. Cells were fixed 10 h p.i. and stained for LAMP1 and HA-tag. DAPI was used for DNA staining (nuclei and S. Typhimurium). b, Quantifications of (a), 100 infected cells assessed for presence of SIFs; P value was calculated using one-way analysis of variance (ANOVA) (n = 3). c, Time course of SopD2 expression in infected HeLa cells. Western blot analysis comparing SopD2-HA expressed from its native promoter (pACYC184 backbone) with SopD2-UltraID-HA expressed from a synthetic constitutive promoter (pCON-D) at 3–23 h post-infection. (TIF) [file ppat.1013507.s001.tif]

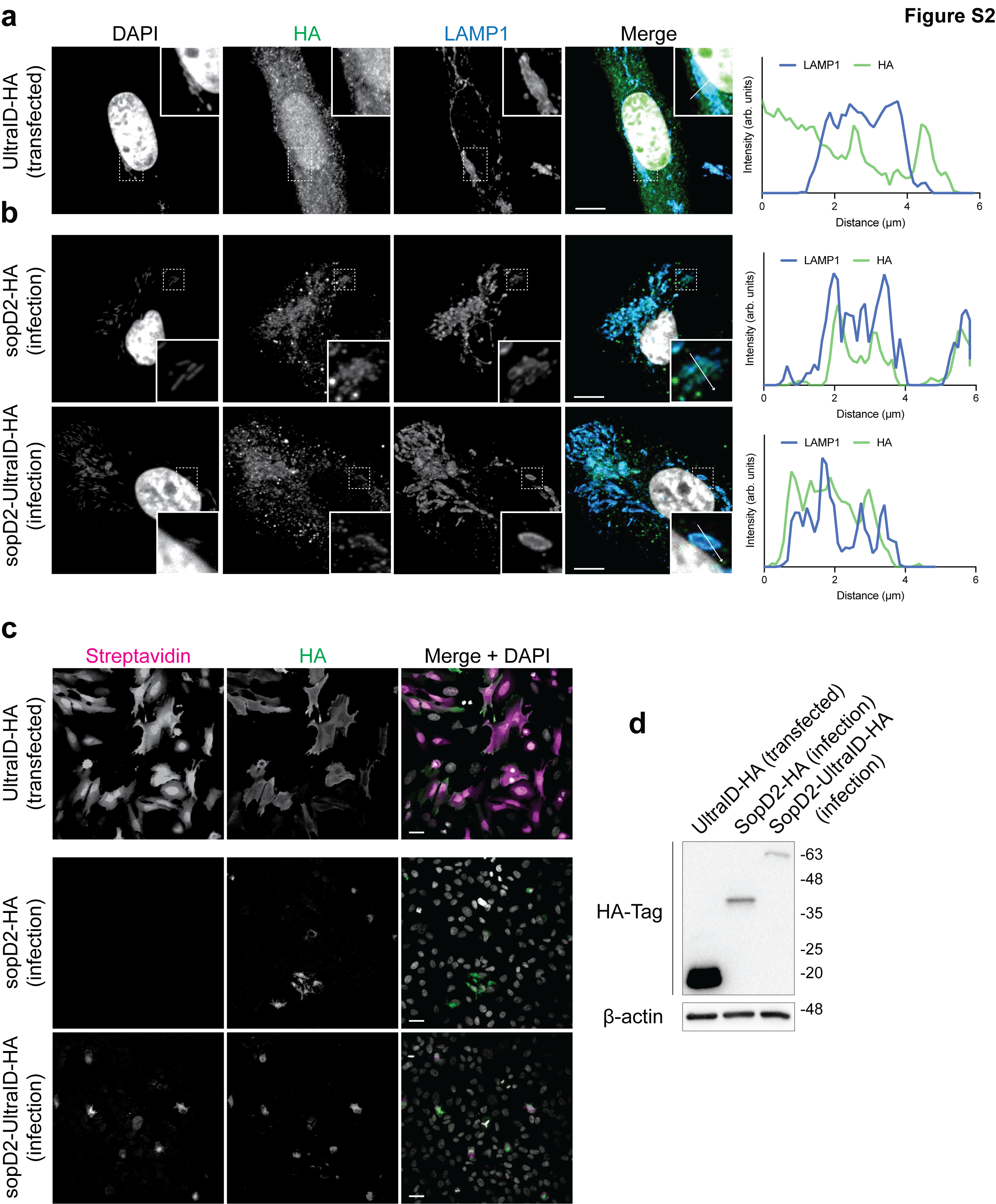

Supplement: S2 Fig — Representative images are shown and the associated scale bars for fluorescence images indicate 10 μm. a, HeLa cells were transfected with UltraID-2HA and infected with S. Typhimurium. Cells were fixed 23 h p.i. and immunostained for LAMP1 and HA-tag. DAPI was used for DNA staining (nuclei and S. Typhimurium). The line plot profile corresponds to the white arrow in the inset of the merged image. b, HeLa cells were infected with ΔsopD2 SL1344 expressing SopD2-HA or ΔsopD2 SL1344 expressing SopD2-UltraID-HA. Cells were fixed 23 h p.i. and immunostained for LAMP1 and HA-tag. DAPI was used for DNA staining (nuclei and S. Typhimurium). The line plot profiles correspond to the white arrows in the insets of the merged images. c, HeLa cells were transfected and infected or infected as described in (a) and (b). Cells were fixed 23 h p.i. and stained for HA-tag and Biotin (Streptavidin 568 probe). d, Western blot of infected host cell lysate. HeLa cells were transfected with UltraID-2HA and infected with S. Typhimurium or infected with ΔsopD2 SL1344 expressing SopD2-HA or ΔsopD2 SL1344 expressing SopD2-UltraID-HA. Cells were harvested 23 h p.i. (TIF) [file ppat.1013507.s002.tif]

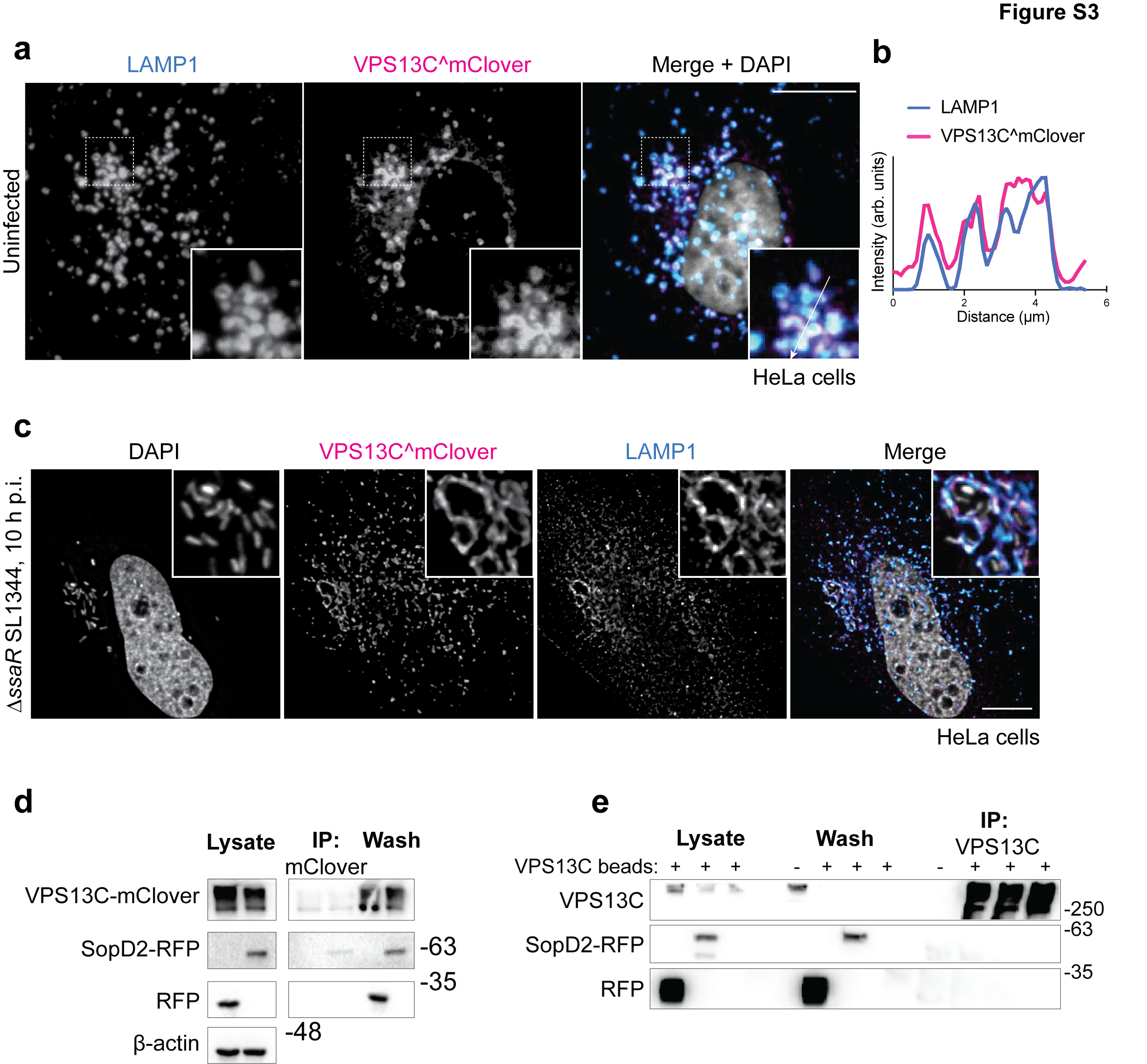

Supplement: S3 Fig — Representative images are shown and the associated scale bars for fluorescence images indicate 10 μm. a, HeLa cells transfected with VPS13C^mClover and immunostained for LAMP1. VPS13C signal was boosted with a GFP antibody. DAPI was used for DNA staining (nuclei and S. Typhimurium). b, Line plot profile of the white arrow in the inset of the merged images in (a). c, HeLa cells transfected with VPS13C^mClover and infected with ΔssaR SL1344. Cells were fixed 10 h p.i. and immunostained for LAMP1. VPS13C signal was boosted with a GFP antibody. DAPI was used for DNA staining (nuclei and S. Typhimurium). d, Immunoprecipitation of VPS13C^mClover from HeLa cells transfected with SopD2-RFP using GFP-trap beads, followed by western blotting for SopD2-RFP. e, Immunoprecipitation of endogenous VPS13C from HeLa cells transfected with SopD2-RFP using anti-VPS13C antibody–conjugated protein G beads, followed by western blotting for SopD2-RFP. (TIF) [file ppat.1013507.s003.tif]

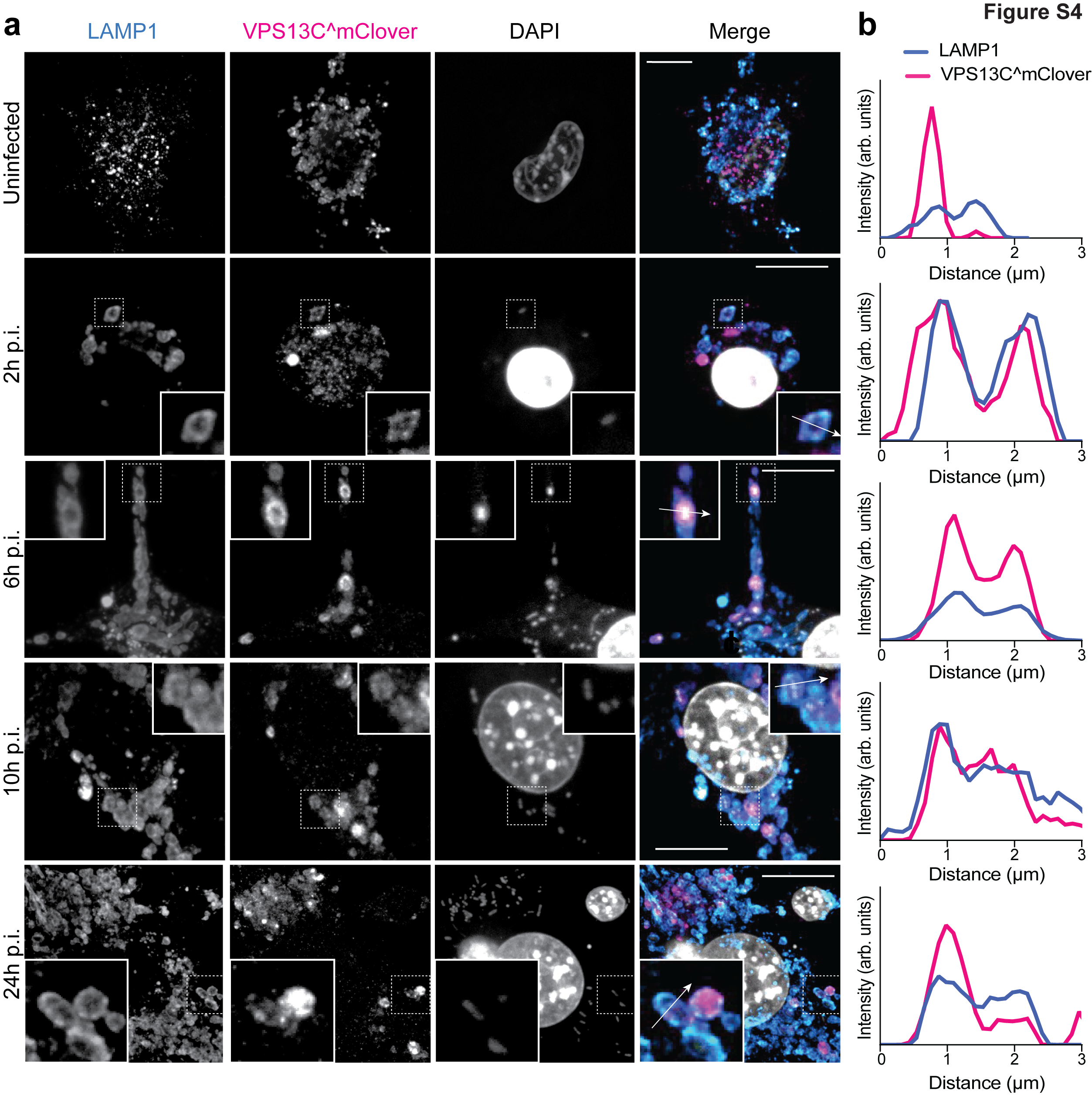

Supplement: S4 Fig — Representative images are shown and the associated scale bars for fluorescence images indicate 10 μm. a, RAW 264.7 macrophages were transfected with VPS13C^mClover by electroporation and infected with S. Typhimurium. Cells were fixed at 2 h, 6 h,10 h or 24 h p.i. and immunostained for LAMP1. VPS13C signal was boosted with a GFP antibody. DAPI was used for DNA staining (nuclei and S. Typhimurium). b, Line plot profile of the white arrow in the inset of the merged images in (a). (TIF) [file ppat.1013507.s004.tif]

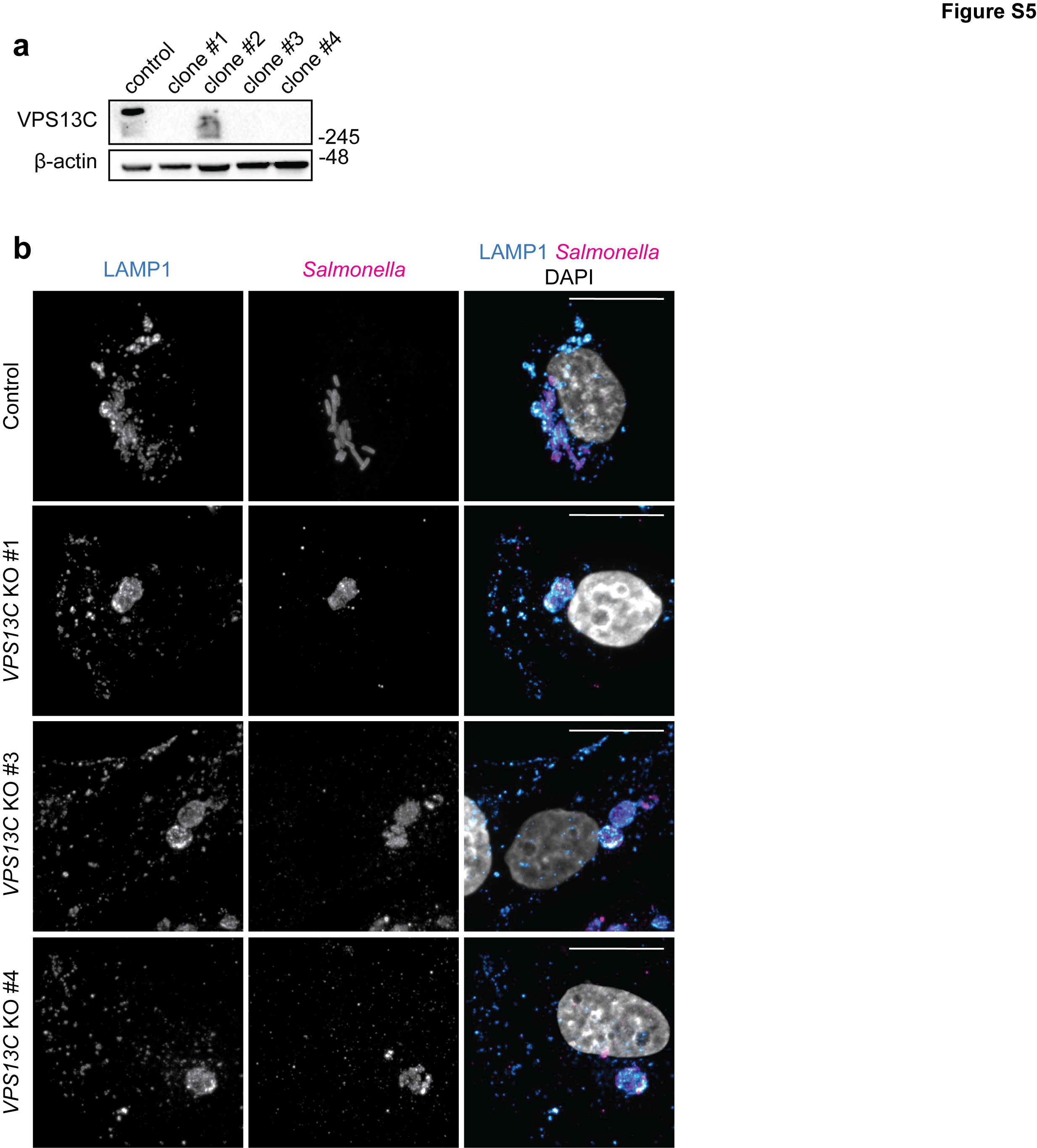

Supplement: S5 Fig — Representative images are shown, and the associated scale bars for fluorescence images indicate 10 μm. a: Three different gRNAs and a combination of gRNAs [1–3] were tested for knockout of VPS13C in HeLa cells. gRNA 3 and the combination of gRNAs [1–3] were sufficient for knockout of VPS13C in HeLa cells. Western blotting was performed to confirm gene knockout. b, Single cell selection was performed on HeLa cells transfected with a combination of gRNAs [1–3]. Clones were expanded and Western blotting was performed to confirm gene knockout. Knockout of VPS13C was confirmed in clones #1,#3 and #4. c, VPS13C KO clones #1,#3 and #4 and control cells were infected with WT SL1344 and immunostained for LAMP1 and Salmonella. (TIF) [file ppat.1013507.s005.tif]

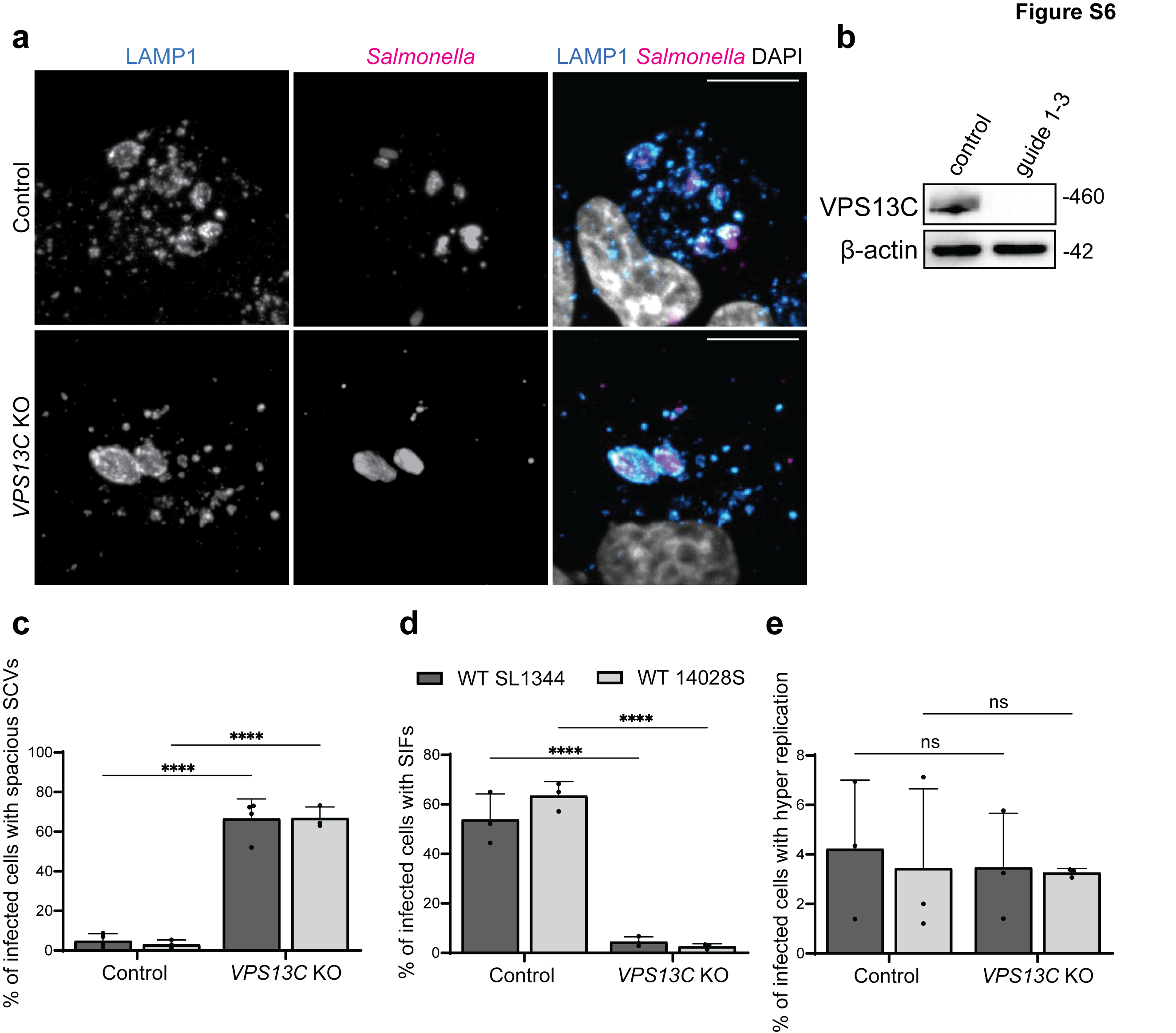

Supplement: S6 Fig — Representative images are shown, and the associated scale bars for fluorescence images indicate 10 μm. a: VPS13C KO U2-OS and control cells infected with S. Typhimurium and immunostained for LAMP1 and Salmonella. b: A combination of gRNAs [1–3] was used for sufficient knockout of VPS13C in U2-OS cells. VPS13C KO U2-OS and control cells were expanded after single-cell selection. Western blotting was performed to confirm gene knockout. Membranes were probed for VPS13C and actin. c,d,e: VPS13C KO HeLa cells and control cells were infected with WT SL1344 and WT 14028S. Quantifications of (c,d,e): 100 infected cells were assessed for the presence of multi-bacterial SCVs (c), SIFs (d), and hyper-replication (e). The averages ± standard deviations for three independent experiments are shown. P values were calculated using two-way ANOVA (n = 3). (TIF) [file ppat.1013507.s006.tif]

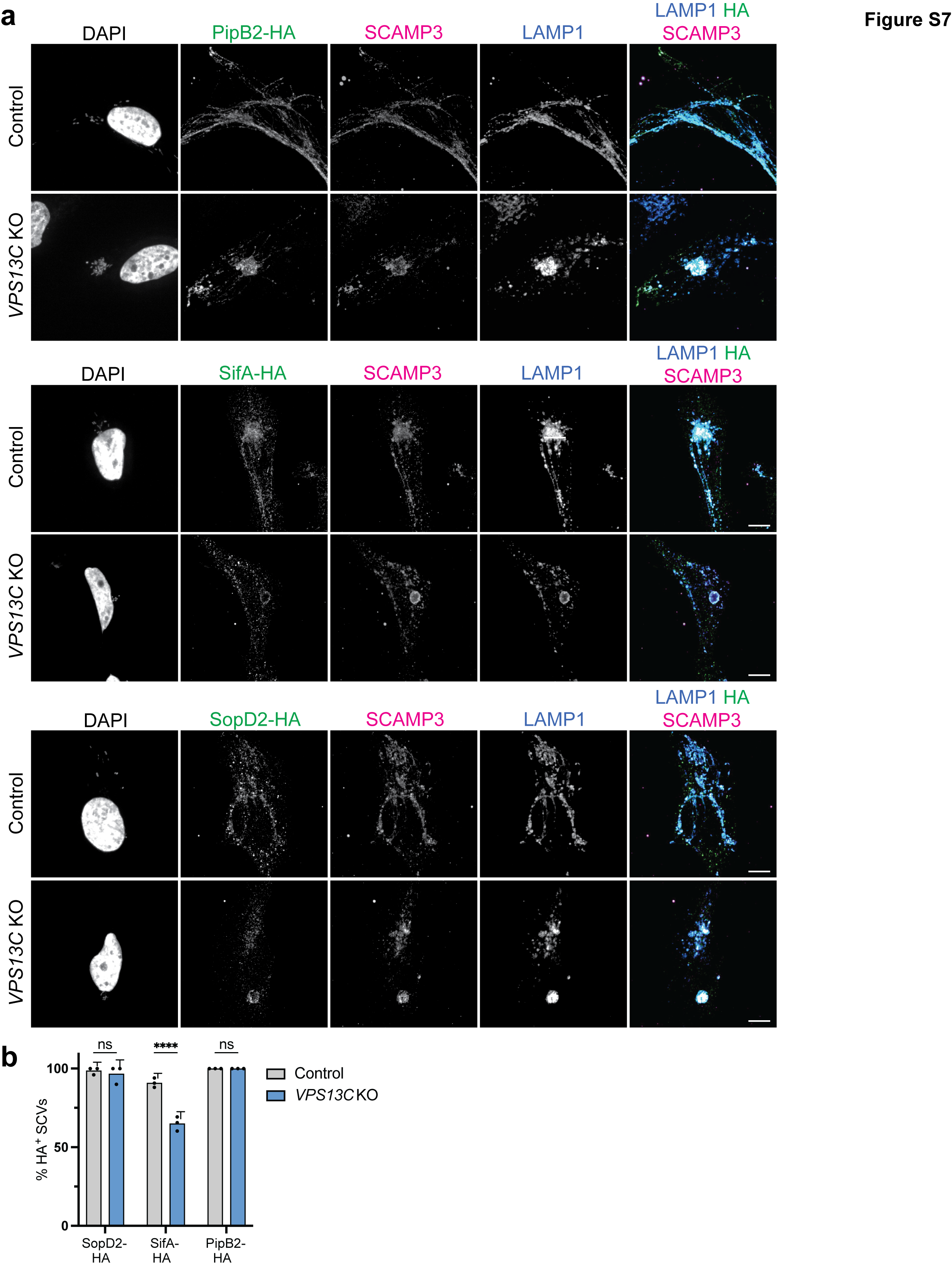

Supplement: S7 Fig — Representative images are shown and the associated scale bars for fluorescence images indicate 10 μm. a, VPS13C KO HeLa cells and control cells were infected with ΔpipB2 SL1344 expressing PipB2-HA, ΔsifA SL1344 expressing SifA-HA, or ΔsopD2 SL1344 expressing SopD2-HA. Cells were fixed 10 h p.i. and stained for LAMP1, SCAMP3 and HA-tag. DAPI was used for DNA staining (nuclei and S. Typhimurium). b, Quantification of (a): a minimum of 50 cells were assessed for the presence of HA signal (PipB2-HA, SifA-HA or SopD2-HA) on SCVs. The averages ± standard deviations for three independent experiments are shown. P values were calculated using two-way ANOVA (n = 3). (TIF) [file ppat.1013507.s007.tif]

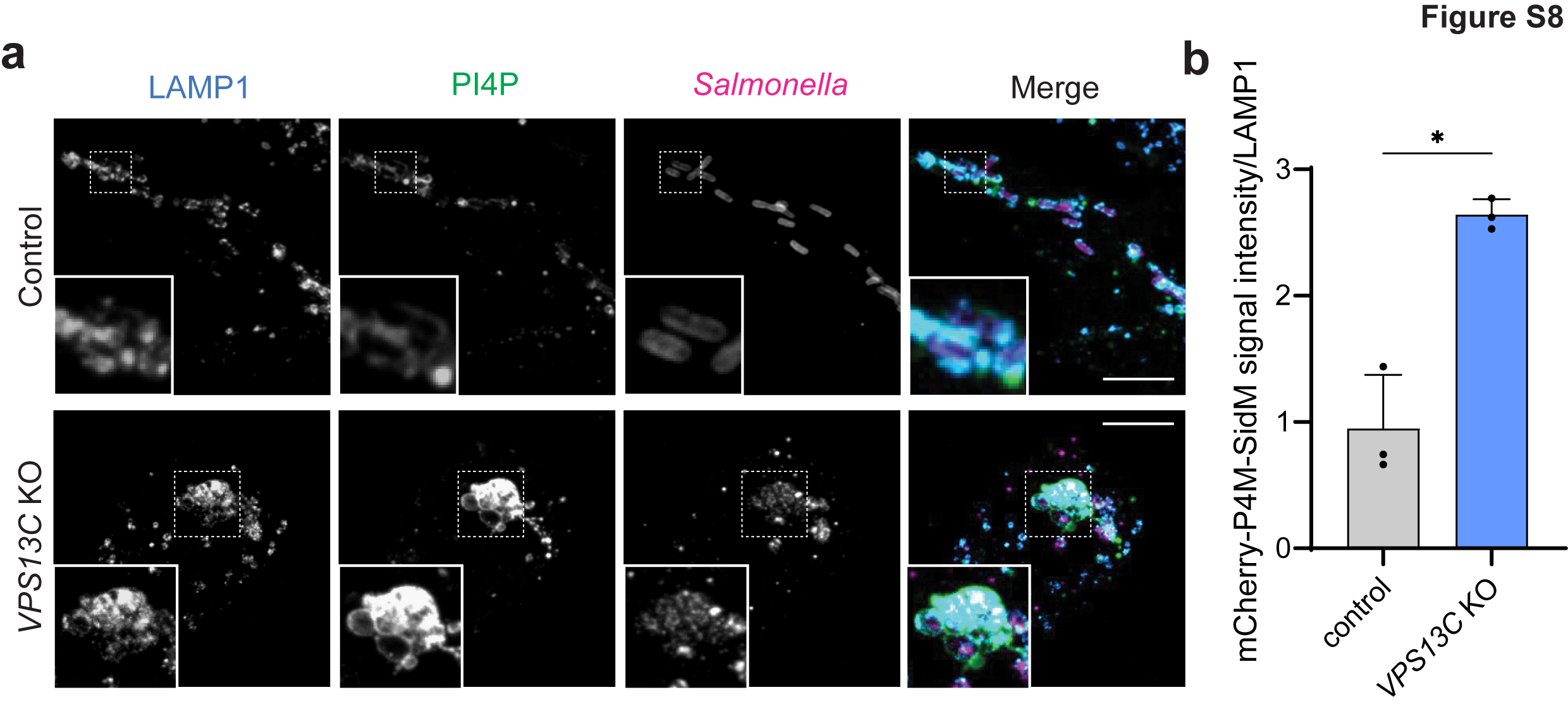

Supplement: S8 Fig — a, VPS13C KO HeLa cells and control cells were transfected with mCherry-P4M-SidM and infected with S. Typhimurium. Cells were fixed 10 h p.i. and immunostained for LAMP1 and Salmonella. b, Quantification of (a). mCherry-P4M-SidM intensity at the SCV was normalized to LAMP1 for at least 30 SCVs per condition per experiment (n = 3). Conditions were compared with a two-tailed ratio paired t-test. (TIF) [file ppat.1013507.s008.tif]

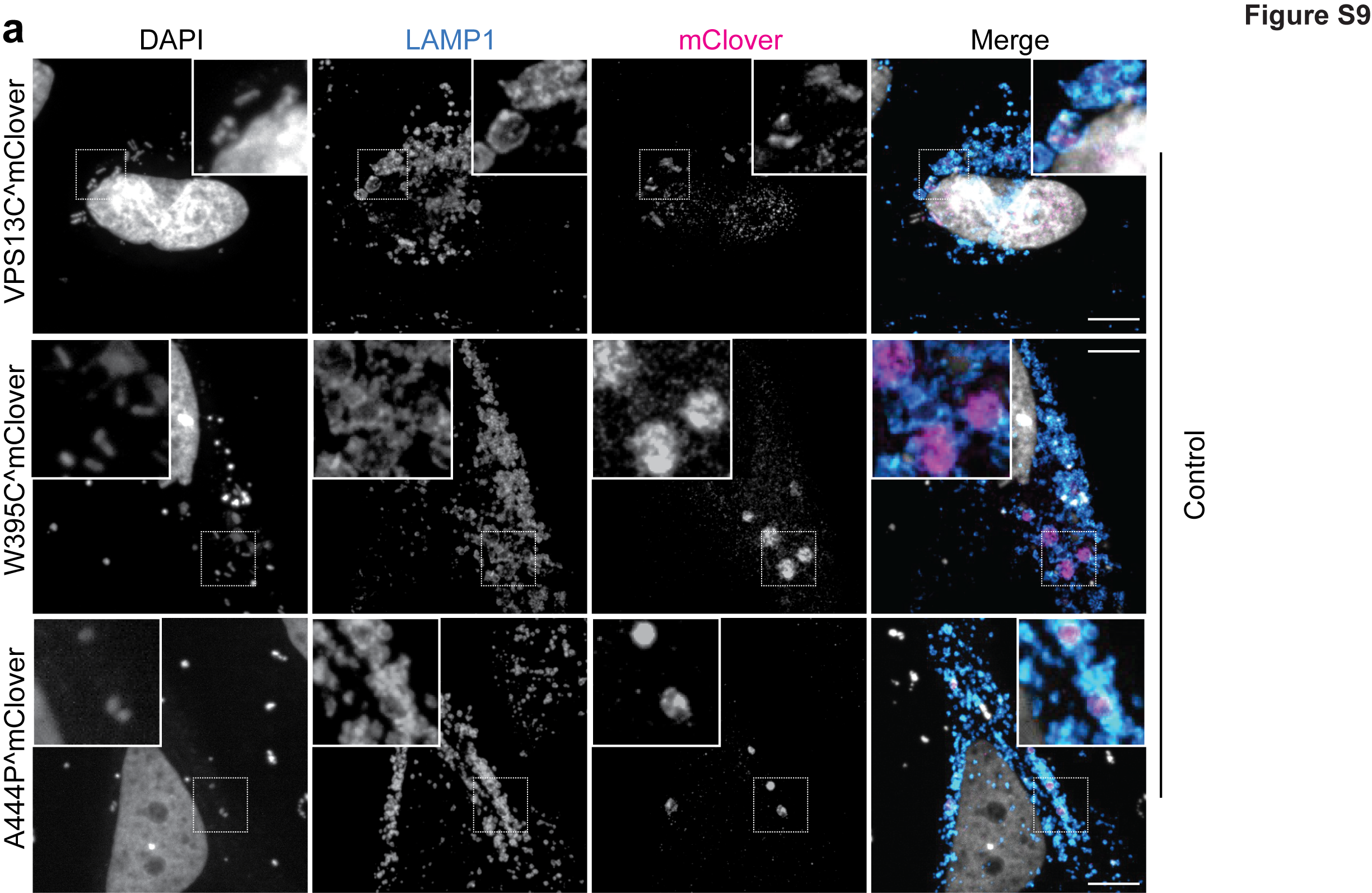

Supplement: S9 Fig — Representative images are shown and the associated scale bars for fluorescence images indicate 10 μm. a, Control HeLa cells were transfected with VPS13C^mclover, A444P-VPS13C, W395C-VPS13C or control plasmid and infected with S. Typhimurium. Cells were fixed 10 h p.i. and immunostained for LAMP1. VPS13C signal was boosted by staining with a GFP antibody. DAPI was used for DNA staining (nuclei and S. Typhimurium). (TIF) [file ppat.1013507.s009.tif]

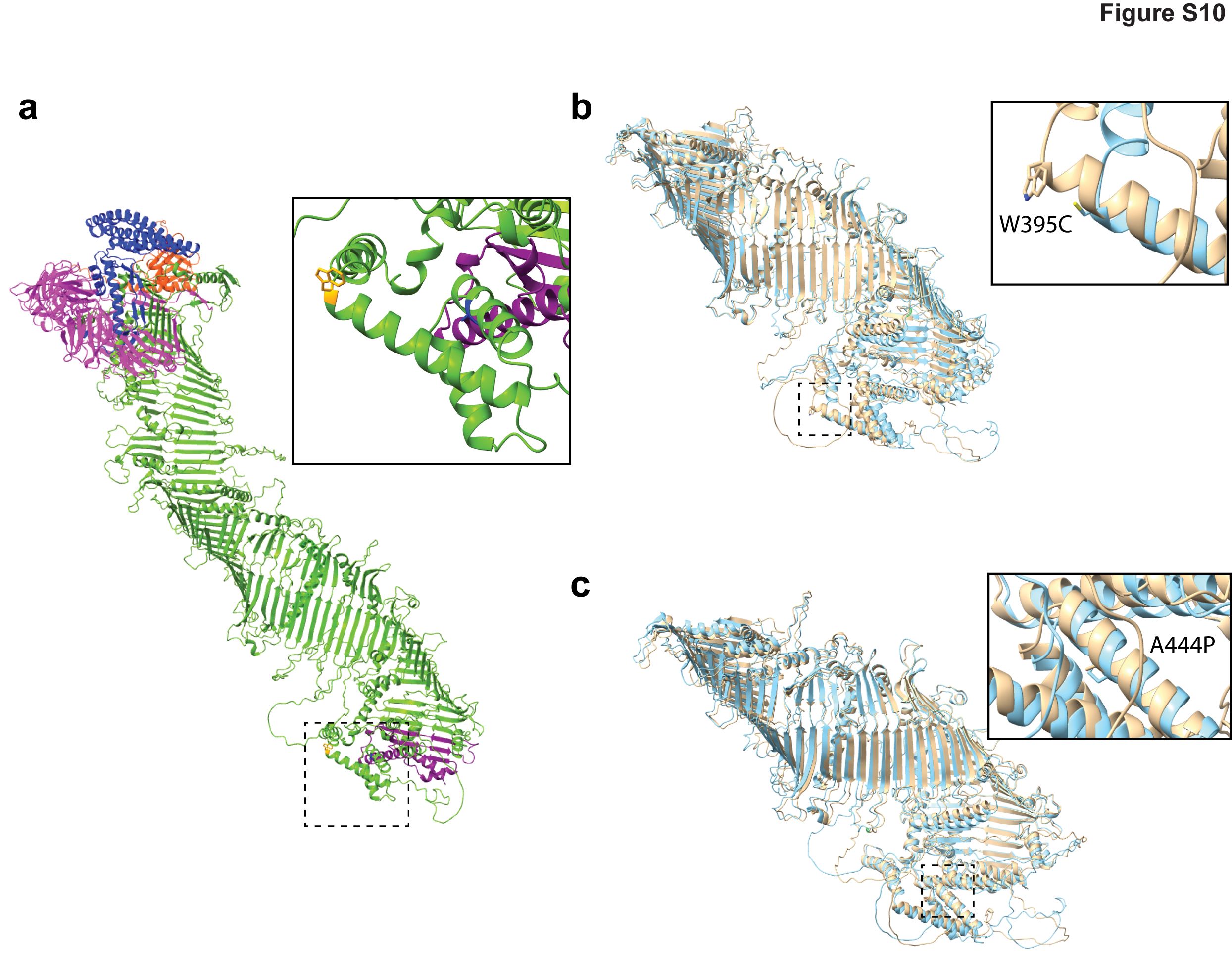

Supplement: S10 Fig — a, The structure of VPS13C predicted by AlphaFold2 [47]. The major domains are indicated as follows: Chorein motif, purple; WD40 modules, magenta; DH-Like domain, blue; Pleckstrin homology domain, orange. The inset is a magnification of the boxed region, which contains the amino acids W395 and A444 (depicted in yellow and blue sticks, respectively). b, AlphaFold3 predicted structures of WT (tan) and W395C (teal) VPS13C (aa 1–1860). Inset is a magnification of the boxed region and depicts stick representations of W395 and C395. c, AlphaFold3 predicted structures of WT (tan) and A444P (teal) VPS13C (aa 1–1860). Inset is a magnification of the boxed region and depicts stick representations of A444 and P444. All AlphaFold3 structural predictions were generated using the same seed and only amino acids 1–1,860, similar to the strategy used by Cai et al. [47]. The Matchmaker tool (Needleman-Wunsch algorithm) in ChimeraX (version 1.10) was used for the structural alignments using the top ranked AlphaFold3 model for each point mutant. (TIF) [file ppat.1013507.s010.tif]
